# 2D-3D integration of hexagonal boron nitride and a high-κ dielectric for ultrafast graphene-based electro-absorption modulators

**DOI:** 10.1038/s41467-021-20926-w

**Published:** 2021-02-16

**Authors:** Hitesh Agarwal, Bernat Terrés, Lorenzo Orsini, Alberto Montanaro, Vito Sorianello, Marianna Pantouvaki, Kenji Watanabe, Takashi Taniguchi, Dries Van Thourhout, Marco Romagnoli, Frank H. L. Koppens

**Affiliations:** 1 ICFO—Institut de Ciències Fotòniques, The Barcelona Institute of Science and Technology, Castelldefels (Barcelona), 08860 Spain; 2 Dipartimento di Fisica “E. Fermi”, Università di Pisa, Pisa, 56127 Italy; 3 Consorzio Nazionale per le Telecomunicazioni (CNIT), Photonic Networks and Technologies National Laboratory, Pisa, 56124 Italy; 4 Department of 3D and Silicon photonics systems, Imec, Leuven, 3001 Belgium; 5 Research Center for Functional Materials, National Institute for Materials Science, Tuskuba, 305-0044 Japan; 6 International Center for Materials Nanoarchitectonics, National Institute for Materials Science, Tsukuba, 305-0044 Japan; 7 Photonics Research Group, Department of Information Technology, Ghent University-IMEC, Gent, 9000 Belgium; 8 ICREA—Institució Catalana de Recerca i Estudis Avançats, Barcelona, 08010 Spain

**Keywords:** Nanoscience and technology, Graphene

## Abstract

Electro-absorption (EA) waveguide-coupled modulators are essential building blocks for on-chip optical communications. Compared to state-of-the-art silicon (Si) devices, graphene-based EA modulators promise smaller footprints, larger temperature stability, cost-effective integration and high speeds. However, combining high speed and large modulation efficiencies in a single graphene-based device has remained elusive so far. In this work, we overcome this fundamental trade-off by demonstrating the 2D-3D dielectric integration in a high-quality encapsulated graphene device. We integrated hafnium oxide (HfO_2_) and two-dimensional hexagonal boron nitride (hBN) within the insulating section of a double-layer (DL) graphene EA modulator. This combination of materials allows for a high-quality modulator device with high performances: a ~39 GHz bandwidth (BW) with a three-fold increase in modulation efficiency compared to previously reported high-speed modulators. This 2D-3D dielectric integration paves the way to a plethora of electronic and opto-electronic devices with enhanced performance and stability, while expanding the freedom for new device designs.

## Abstract (web summary)

Here, three-dimensional hafnium oxide and two-dimensional hexagonal boron nitride are integrated in the insulating section of double-layer graphene optical modulators, leading to a maximum bandwidth of 39 GHz and enhanced modulation efficiency.

## Introduction

Broadband optical modulators with ultra-high-speed, low-drive voltage, and hysteresis-free operation are key devices for next-generation datacom transceivers^1^. Although Si photonics is nowadays a prime candidate to fulfill these requirements^2,3^, graphene is rapidly becoming a major contender in several optoelectronic applications, such as ultrafast modulators^4,5^ and silicon-integrated photodetectors^6,7^. Graphene-based modulators have already proven broadband optical bandwidth^1^, high speed^8,9^, relatively high modulation efficiencies^10^, and temperature stability^8^. These devices are all based on complementary metal–oxide–semiconductor (CMOS)-compatible material^7,10–13^, where CMOS design and fabrication techniques can be further leveraged to decrease costs. However, graphene-based modulators are yet to demonstrate all operation requirements at once. More specifically, EA graphene modulators struggle to show high-speed and high modulation efficiencies simultaneously^14^. This bottleneck is mostly due to the weak graphene/dielectric combination and the limited quality of graphene.

Unlike Si technology, where high-*κ* dielectrics lie at the core of its success, 2D dielectrics are hindering the development of graphene- and other 2D-based electronics and optoelectronic devices^1,13,15^ and are clearly outperformed by traditional 3D high-*κ* dielectrics. This underperforming 2D-dielectric/graphene combination deepens even further the fundamental trade-off between speed and modulation efficiency inherent to the double-layer (DL) modulators^14^. In the DL architecture, the overlapped top and bottom graphene electrodes act as a capacitor (*C*). The larger the *C*, the higher the modulation efficiency. On the other hand, the speed of the modulator defined as *f*_3dB_ = 1/(2*πRC*) is inversely proportional to *C* (*R* being the total resistance). In this framework, the quality of graphene appears as a valid turnaround to overcome this fundamental limitation. High electron mobility is expected to minimize the overall resistance and reduce the insertion loss (IL)^1,9^, thus increasing the bandwidth and the extinction ratio (ER). However, the quality of graphene is very sensitive to its environment, e.g., the dielectric to encapsulate it. Indeed, no graphene/dielectric combination has been able to ensure high charge carrier mobilities and low levels of residual doping in existing graphene waveguide-coupled modulators^16^. The growth of nonlayered (i.e., 3D) dielectrics, e.g., aluminum oxide (Al_2_O_3_), silicon nitride (${\rm{SiN}}$), or HfO_2_ directly on top of graphene leads to low electronic mobility^16–18^ and/or inhomogeneous doping^19^.

In this work, we demonstrate the 2D–3D integration of hBN and HfO_2_ within the dielectric section of a DL graphene EA modulator. This dielectric combination enhances the capacitance of the EA modulators without compromising their robustness against high voltages and preserves the high mobility and low doping of intrinsic graphene. As a result, we achieved a static and dynamic (at 40 Gbps) modulation efficiency as high as 2.2 and 1.49 dBV^−1^, respectively, a *f*_3dB_ bandwidth of ~39 GHz, and a device footprint of 60 μm × 0.45 μm ≈ 27 μm^2^ (neglecting the metal pads and graphene leads). Moreover, the hBN–HfO_2_–hBN-based devices show a symmetric and nearly hysteresis-free operation. The larger breakdown voltage of this 2D–3D dielectric, even beyond the full transparency regime (i.e., Pauli blocking), increases the ER and reduces the IL of the modulators.

## Results

**Fig. 1:**
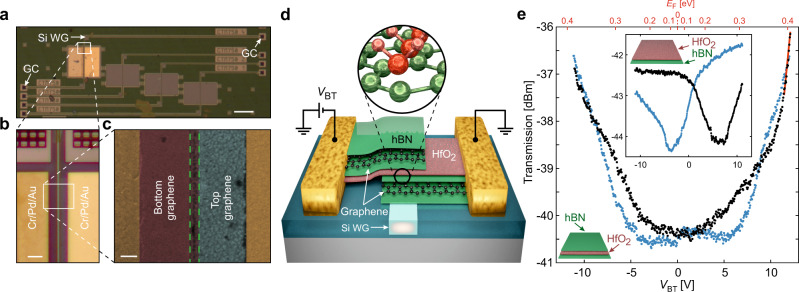
Device geometry and static characterization. **a** Optical image of a photonic device consisting of two grating couplers (GC), a silicon optical waveguide (Si WG), and an hBN–HfO_2_–hBN-based graphene EA modulator on top (see zoom-in optical (panel **b**) and scanning electron microscope (SEM) (panel **c**) images for details). In panel **c**, the metal contacts are yellow/brown and the bottom and top graphene electrodes violet and light blue, respectively. The core of the waveguide is highlighted by the green dashed lines. The white scale bars in panel a, b, and c are 100, 5, and 1 μm, respectively. **d** Electrical connections and schematic cross-section of an EA modulator with an hBN–HfO_2_–hBN dielectric. The top and bottom graphene electrodes are fully encapsulated by hBN (in green) protecting both graphene electrodes from the out-of-plane dangling bonds typical of 3D oxide materials, e.g., HfO_2_ (in red). See inset for a molecular representation. **e** Transmission curves as a function of the voltage between the bottom and top graphene electrodes (*V*_BT_ axis, bottom) and the Fermi energy at the graphene electrodes (*E*_F_ axis, top) for the EA modulator in panel a with an hBN–HfO_2_–hBN dielectric (see sketch). The 1550 nm excitation power was set to 0 dBm. The forward and backward voltage sweeps (black and blue, respectively) show no major hysteresis compared to a modulator with an hBN–HfO_2_ dielectric (see inset). The red line is a linear fit to the forward voltage sweep within a 0.5 V voltage span (extracted slope: 2.2 dBV^−1^).

The EA modulators were fabricated on top of a photonic structure^20^ formed by two grating couplers^21^ feeding light in and out of an optical waveguide (Fig. 1a). The 750-nm-wide waveguide for the device in Fig. 1 was designed to support a single transverse–magnetic (TM) optical mode^20^ (see Supplementary Note 3). The presented DL graphene modulators were built, with hBN-encapsulated graphene top and bottom electrodes (Fig. 1d). The hBN–graphene–hBN stacks have been fabricated following state-of-the-art fabrication techniques^22,23^. This ensured low levels of doping and high charge carrier mobilities. We characterized the quality of the resulting modulators (see Supplementary Notes 2 and 6) and extracted a carrier density-independent mobility as high as 30,000 cm^2^ V^−1^ s^−1^ at room temperature^23^ (see Supplementary Note 2).

Although hBN-encapsulated graphene devices have allowed for device designs with unprecedented functionalities^24–26^ and improved performance^23^, such layered dielectric material typically contains impurities and/or crystal defects leading to low breakdown voltages^27,28^. Moreover, the dielectric permittivity of hBN is rather low compared to existing high-*κ* dielectrics^29^, with a value close to that of SiO_2_ (*ϵ*_r_ ~ 4). This low dielectric constant and reduced breakdown voltage (see Supplementary Note 5) compromises not only the power consumption and the ability to reach high modulation efficiencies at reasonably low drive voltages but also limits the IL and the ER of the modulators^1,9^. We thus integrate HfO_2_, a high-*κ* dielectric material, within the hBN-encapsulated graphene electrodes (see the sketch in Fig. 1d).

With such hBN–HfO_2_–hBN dielectric arrangement, graphene remains isolated from HfO_2_, shielded away from any possible out-of-plane dangling bonds of the 3D oxide material (see inset of Fig. 1d for the molecular representation of the 2D–3D dielectric interface). More importantly, the hBN–graphene interfaces remain atomically sharp and clean^22,23,30^. This nanoscale control of the interfaces brings further advantages to real-world EA graphene modulators, like a symmetric and hysteresis-free operation. This is directly visible in the transmission curves as a function of the applied voltage *V*_BT_ or, alternatively, as a function of the Fermi energy *E*_F_ at the graphene electrodes (see the bottom and top axis in Fig. 1e and Supplementary Note 9). Both forward and backward voltage sweeps (black and blue traces, respectively) show minor hysteresis and appear symmetric with respect to the charge neutrality point. For comparison, a device fabricated with a HfO_2_–hBN dielectric shows no overlap between the forward and backward sweeps (inset of Fig. 1e). This strong hysteresis is nonetheless expected for this HfO_2_–hBN modulator since, in that case, the top graphene electrode is in direct contact with HfO_2_. The hBN–HfO_2_–hBN modulator device exhibits a modulation efficiency as high as ~2.2 dBV^−1^ within a 0.5 V voltage span (see red linear fit to the data in Fig. 1e). Considering the length of our modulator (~60 μm), we obtain a normalized static modulation efficiency of ~0.037 dBV^−1^ μm^−1^, a threefold increase compared to previously reported high-speed graphene EA modulators^9^.

**Fig. 2:**
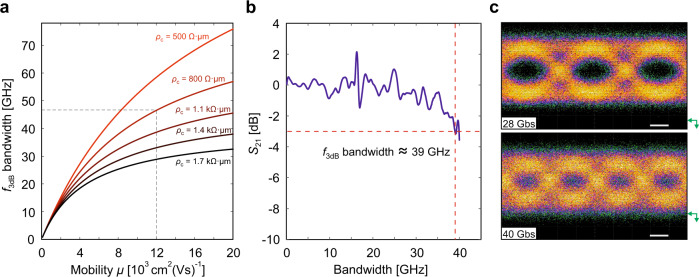
Dynamic characterization. **a** *f*_3dB_ bandwidth as a function of the charge carrier-dependent mobility (*μ*) and the contact resistivity (*ρ*_c_) calculated for a device with the same geometry and dielectric combination as the device in Fig. 1 (see Supplementary Note 11). The dashed lines indicate the expected *f*_3dB_ ~46 GHz at *μ* ~12,000 cm^2^ V^−1^ s^−1^ (evaluated at *V*_BT_ = 10.4 V, refer to panel **b**). **b** Measured electro-optical *S*_21_ frequency response of the EA modulator at *V*_BT_ = 10.4 V and *V*_AC_ = 200 mV, without de-embedding, i.e., including the contributions of the setup and photodetector (see Supplementary Note 12). **c** In all, 2^31^ − 1 pseudo-random binary sequence non-return-to-zero eye diagram at 28 and 40 Gbps. The EA modulator is d.c. biased at *V*_BT_ = 11 V and driven by a *V*_AC_ = 3.5 V peak-to-peak RF signal. The eye diagram measured at 40 Gbps has a 5.2 dB ER and a 2.28 dB signal-to-noise ratio (SNR). The green arrows indicate the 0 *W* baseline, and the white scale bar corresponds to 10 ps.

With such a high static modulation efficiency (Fig. 1), one might expect the device speed to be compromised^14^. However, the high mobility of the hBN-encapsulated graphene is expected to increase the bandwidth. This is visible in Fig. 2a, where we calculated the *f*_3dB_ bandwidth as a function of the charge carrier-dependent mobility (*μ*) and contact resistivity (*ρ*_c_) for a graphene modulator with the same geometry and dielectric combination as the device in Fig. 1 (see Supplementary Note 11). As observed, the graphene mobility and the contact resistivity have a major influence on the modulator speed. Considering the mobility *μ* ≈ 12,000 cm^2^ V^−1^ s^−1^ (evaluated at *V*_BT_ = 10.4 V) and the contact resistivity *ρ*_c_ ≈ 800 Ω μm achieved experimentally (see Supplementary Notes 4 and 11), we expect a bandwidth of *f*_3dB_ ~ 46 GHz (dashed lines in Fig. 2a). To confirm this value experimentally, we measured the electro-optical (EO) bandwidth of the device in Fig. 1 at a DC voltage *V*_BT_ = 10.4 V and a peak-to-peak voltage *V*_AC_ = 200 mV (Fig. 2b). The bandwidth of the measured device attains *f*_3dB_ ≈ 39 GHz (without de-embedding, see Supplementary Note 13). This value is close to the capabilities of our setup, limited to 40 GHz by the vector network analyzer and the RF probes (see Supplementary Note 12). Even though the measured *f*_3dB_ does not reach the expected *f*_3dB_ ~ 46 GHz (Fig. 2a), possibly due to an increased contact resistivity of the measuring device (see Supplementary Note 11), this is still the highest *f*_3dB_ bandwidth among all graphene-based modulators reported so far^8,9,11,12,31,32^.

The high-speed operation of our modulator device is also supported by non-return-to-zero eye diagram measurements. The data were obtained through an electrical pattern generator (PG) driving the modulator with a 2^31^ − 1 pseudo-random binary sequence at 28 and 40 Gbps bit-rate (see Supplementary Note 13). The signal was driven by a 3.5-V peak-to-peak voltage while the DC bias was set to 11 V. The device was terminated with a 50 Ω load to avoid reflections due to the impedance mismatch between the PG electrical output and the modulator (when measured at 40 Gbps). Open eye diagrams at 28 and 40 Gbps are shown in Fig. 2c, with an ER as high as 5.2 dB and a signal-to-noise ratio of 2.28 dB for the latter (see Supplementary Note 14 for an eye diagram at 10 Gbps). These results confirm the large modulation efficiency of our hBN–HfO_2_–hBN-based modulator device, even at high speeds, with a dynamic modulation efficiency of 1.49 dBV^−1^ at 40 Gbps^9^.

Like the speed of the modulator, the power consumption understood as the switching energy per bit also benefits from the small footprint of the device. Ignoring the parasitic pad capacitance, we obtain for the modulator in Fig. 1 an energy per bit of $C{({V}_{{\rm{AC}}})}^{2}/4\approx {160}\ {{\rm{fJbit}}}^{-1}$, where *C* = 52 fF is the capacitance between the top and bottom graphene electrodes and *V*_AC_ = 3.5 V the voltage swing^12^. This value of energy per bit is on par with state-of-the-art SiGe technologies^33,34^.

**Fig. 3:**
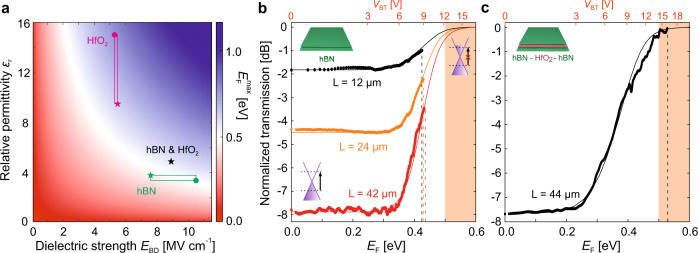
Dielectric breakdown and Pauli blocking operation. **a** Maximum Fermi energy, noted ${E}_{{\rm{F}}}^{\max }$, expected at the graphene electrodes of a graphene modulator with a dielectric’s relative permittivity *ϵ*_r_ and dielectric strength *E*_BD_. All points lying inside the blue-colored region represent a dielectric allowing for Pauli blocking operation (${E}_{{\rm{F}}}^{\max }\,> \, 0.5$ eV, refer to Supplementary Note 1). The red-colored region indicates otherwise (${E}_{{\rm{F}}}^{\max }\,<\, 0.5$ eV). The white band represents the Pauli blocking boundary condition, defined as ${E}_{{\rm{F}}}^{\max }=0.5$ eV. The expected ${E}_{{\rm{F}}}^{\max }$ for HfO_2_ and hBN are represented by the red and green squares, respectively, taking the values of *E*_BD_ and *ϵ*_r_ from literature^28,35–37^ (marked with dots) and our dielectric characterization (marked with stars, see Supplementary Notes 5 and 10). The black star represents the ${E}_{{\rm{F}}}^{\max }=0.57$ eV expected for the hBN–HfO_2_–hBN modulator in Fig. 1e (see Supplementary Note 10). **b**, **c** Normalized transmission as a function of *E*_F_ and *V*_BT_ for modulators with hBN (b) and hBN–HfO_2_–hBN (c) dielectric. The data points are measurements and the solid curves simulations (see Supplementary Notes 1–3 and 10). The vertical dashed lines indicate the ${E}_{{\rm{F}}}^{\max }$ achieved at the dielectric breakdown. The orange-shaded regions show the full transparency range, i.e., Pauli blocking. The top *V*_BT_ axis in panel b is for the 42 μm-long device only (see Supplementary Note 7 for the other hBN devices). The graphene Dirac cones in panel b show the absorption and Pauli blocking processes at low and high Fermi energies, respectively.

To directly compare modulators with different dielectrics, it is more convenient to compare the transmission as a function of *E*_F_ (see the *E*_F_ axis in Figs. 1e and 3b and c) since *E*_F_ already considers the thickness and the relative permittivity of the dielectric (see Supplementary Note 7). Operating the modulators at high *E*_F_ enhances both ER and IL, with the ER (IL) increasing (decreasing) as a function of *E*_F_^9^. In the full transparency regime (Pauli blocking, see Supplementary Note 1), the ER is maximized and the IL is expected to become nearly zero for high-quality graphene^1,9^ (see Supplementary Note 10). It is thus crucial to determine which dielectric materials facilitate Pauli blocking operation. Figure 3a illustrates the expected maximum *E*_F_,

1 $${E}_{{\rm{F}}}^{\max }={\hslash} {v}_{{\rm{F}}}\sqrt{\pi {\epsilon }_{0}{\epsilon }_{{\rm{r}}}{E}_{{\rm{BD}}}/q},$$

as a function of the relative permittivity (*ϵ*_r_) and dielectric strength (*E*_BD_) of any given dielectric. The square boxes in Fig. 3a enclose the expected ${E}_{{\rm{F}}}^{\max }$ for the HfO_2_− and hBN-based modulators (in red and green, respectively) and the black star represents the ${E}_{{\rm{F}}}^{\max }=0.57$ eV expected for the hBN–HfO_2_–hBN modulator of Fig. 1e (see Supplementary Note 10). The boundaries of the boxes are taken from literature^28,35–37^ (marked with dots) and from our dielectric characterization (marked with stars, see Supplementary Notes 5 and 10). All dielectric materials fulfilling ${E}_{{\rm{F}}}^{\max }\,> \, 0.5$ eV (see white fringe in Fig. 3a) allow full transparency, i.e., Pauli blocking. The comparison in Fig. 3a highlights the advantages of the hBN–HfO_2_–hBN dielectric (black star), achieving higher *E*_F_ values than the hBN dielectric while equally preserving the intrinsic qualities of graphene.

These results are confirmed by the transmission traces in Fig. 3b, c. None of the hBN-based modulators were able to withstand Pauli blocking operation (orange-shaded region Fig. 3b), all breaking their hBN dielectric at a similar ${E}_{{\rm{F}}}^{\max }\approx 0.4$ eV (see vertical dashed lines in Fig. 3b and Supplementary Notes 7 and 8). Even though these hBN-based modulators were too fragile, we obtained modulation efficiencies as high as 0.3, 1.3, and 2 dBV^−1^ for device lengths *L* = 12, 24, and 42 μm, respectively. Once normalized by its length, we obtain 0.025, 0.054, and 0.047 dBV^−1^ μm^−1^. These results exceed the state-of-the-art modulation efficiency of 0.038 dBV^−1^ μm^−1^^10^. Still, the premature hBN breakdown compromises the ER and the IL. Indeed, the measured ER = 0.75, 2.3, and 4.9 dB (data points in Fig. 3b) is far from the simulated ER = 1.8, 4.4, and 7.9 dB (solid traces in Fig. 3b) expected for the 12, 24, and 42 μm-long modulators, respectively (for simulations, refer to Supplementary Notes 1–3). Likewise, the measured IL = 1, 2.2, and 3.4 dB are higher than IL ≈0 dB expected for high-mobility graphene modulators^1^ (see the minimum 0-dB normalized transmission, i.e., neglecting the losses from grating couplers and Si waveguide, achieved by the simulation traces in Fig. 3b and Supplementary Note 10).

On the other hand, the second hBN–HfO_2_–hBN modulator device attains the Pauli blocking regime (Fig. 3c), in agreement with the dielectric characterization of hBN–HfO_2_–hBN (Fig. 3a and Supplementary Notes 5 and 10), reaching a maximum Fermi energy of ${E}_{{\rm{F}}}^{\max }\approx 0.54$ eV. The ER and IL improve accordingly, with an ER = 7.8 dB almost twice the value obtained by the hBN-based modulator of comparable length (compare the black and red traces of Fig. 3c, b, respectively) and an IL reaching nearly zero (IL ≈ 0.04 dB in Fig. 3c and Supplementary Note 10). However, being shorter (*L* = 44 μm) than the device in Fig. 1e (*L* = 60 μm), the modulation efficiency is lower (1.3 dBV^−1^ in a 0.5 V span, see Fig. 3c). We note that the hBN–HfO_2_–hBN device of Fig. 1e has a relatively weak measured ER ≈ 4.4 dB and IL ≈7.8 dB (see Supplementary Note 10) due to an overcautious *V*_BT_ = 12.1 V applied voltage (or alternatively *E*_F_ = 0.41 eV). Considering the breakdown capabilities of hBN–HfO_2_–hBN dielectric (black star in Fig. 3a), we evaluated a potential ER ≈ 12 dB and IL ≈ 0.042 dB for this device (see Supplementary Note 10).

## Discussion

**Fig. 4:**
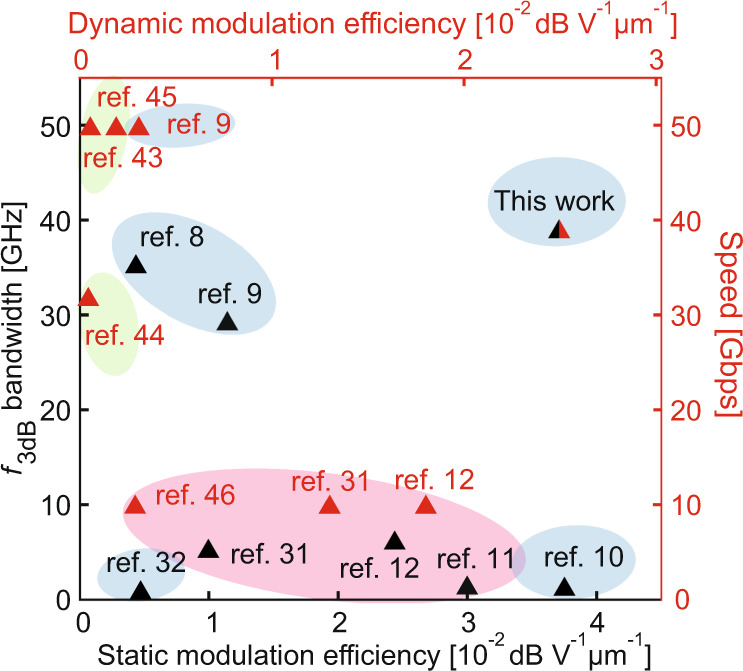
Comparison with other optical modulators. The black and red data points and axis represent the static modulation efficiency as a function of the *f*_3dB_ bandwidth and the dynamic modulation efficiency (extracted from eye diagrams) as a function of the modulation speed, respectively. The red, blue, and green data clouds enclose single-^11,12,31,46^ and double-layer^8–10,32^ graphene and silicon^43–45^ state-of-the-art modulators operating at *λ* = 1.55 μm. Refer to Supplementary Notes 15 and 16 for a more detailed comparison of graphene-based modulators.

Although material platforms like lithium niobate^38^ (LiNbO_3_) or hybrid technologies like Si/indium phosphide^39^ (InP), Si/SiGe^40^, or InGaAlAs^40^ offer outstanding performances in modulator applications, those are either not scalable^38,41^ (LiNbO_3_) or their integration with a CMOS fabrication line remains challenging^40,42^. Nowadays, Si and graphene are envisaged as the most scalable, cost-effective, and CMOS-compatible materials for amplitude modulator applications^1^. To compare our results with state-of-the-art graphene and Si amplitude modulators, both EA and Mach–Zehnder interferometer configurations included, we summarize our results in Fig. 4 and in Supplementary Notes 15 and 16. Figure 4 shows the dynamic modulation efficiency (extracted from the eye diagrams and normalized by the device length and drive voltage) as a function of the modulation speed (red axis and red data point in Fig. 4) and the static modulation efficiency (measured in DC and normalized by the device length), as a function of the *f*_3dB_ bandwidth (black axis and black data point in Fig. 4). To avoid discrepancies due to the different extraction methods, we determine the static modulation efficiency of the compared literature^8–12^ using the same method as in Fig. 1e, i.e., by applying a linear fit within a 0.5 V voltage span. The results highlight the trade-offs between speed and modulation efficiency and stress the advantages of an hBN–HfO_2_–hBN dielectric to obtain large static and dynamic modulation efficiencies even at high speed. As observed, the modulation efficiency typically drops for devices with high speed^8,9^, being our device the only modulator able to operate at high speed with a large static and dynamic modulation efficiency (Fig. 4). These results outperform state-of-the-art graphene and not-yet-commercial silicon-based electroabsorption modulators^43–45^ (see blue/red and green data clouds, respectively in Fig. 4) when considering the modulation efficiency normalized by the length (i.e., footprint). This figure-of-merit is rather an important one since for many envisaged applications (e.g., chip interconnects), multiple modulator devices are expected to coexist on the same chip.

In this work, we demonstrated the advantages of integrating hBN with a 3D high-*κ* dielectric for high-quality graphene-based EA modulators. Compared to traditional oxide sputtering or atomic layer deposition (ALD) growth on top of graphene, the integration of HfO_2_ in-between hBN prevented any damage to the underlying graphene and allowed clean graphene–hBN interfaces. These clean interfaces yielded a symmetric and nearly hysteresis-free operation. Moreover, this 2D–3D integration enabled full transparency while maintaining the high mobility and low doping of intrinsic graphene. More importantly, the hBN–HfO_2_–hBN-based EA modulators were able to reach high modulation speeds with strong modulation efficiencies, overcoming the fundamental limitations of the DL graphene configuration and outperforming state-of-the-art graphene and Si technologies. The compatibility of this hBN–HfO_2_–hBN dielectric with Si and other 2D materials might allow for considerable scaling improvements and greater device functionality in a broad range of graphene- and 2D-based electronic and optoelectronic applications, even beyond graphene-based modulators.

## Methods

### Device fabrication

The Si photonic waveguide with a core cross-section of 750 nm × 220 nm was prepared on the IMEC iSiPP25G silicon-on-insulator platform^20^. For the fabrication of the electroabsorption modulator, the graphene and hBN flakes were exfoliated from highly oriented pyrolytic graphite and hBN crystals, respectively. The bottom hBN–graphene–hBN stacks were prepared by the van der Waals assembly technique^22,23^ and transferred directly onto the Si waveguide separated by a 10 nm spacer of high-quality thermal SiO_2_. The bottom hBN flake (separating the graphene and the SiO_2_ layer) thickness of ~5 nm was chosen to enhance the graphene absorption while isolating the graphene from the rough SiO_2_ substrate. The top hBN has a thickness of ~10 nm. The stack has been etched by reactive ion etching in an oxygen (O_2_) and trifluoromethane (CHF_3_) (4:40 sccm) environment to expose the graphene edge. The bottom stack was then contacted by a 3/15/30 nm Cr/Pd/Au metal combination. The 10 nm hafnium oxide film has been deposited at 250 °C prior depositions of a 2 nm sputtered SiO_2_ seed layer by ALD. Tetrakis-dimethylamido hafnium (TDMAH) (0.4 s purge time) and water vapor (5 s purge time) as precursors have been used in a Savannah G1 system from Cambridge Nanotech. The top hBN–graphene–hBN stack with a 7 nm- and 21 nm-thick bottom and top hBN layers has followed the same fabrication steps as the bottom stack.

## Supplementary information

Supplementary Information

## Data Availability

The data that support the plots within this paper and other findings of this study are available from the corresponding author upon reasonable request.
