# Supplementary material for: 2D-3D integration of hexagonal boron nitride and a high-κ dielectric for ultrafast graphene-based electro-absorption modulators
Source: Nat Commun. 2021 Feb 16;12:1070. doi: 10.1038/s41467-021-20926-w (PMC7887197; doi:10.1038/s41467-021-20926-w)
Supplement: Supplementary file 1 — Supplementary Information [file 41467_2021_20926_MOESM1_ESM.pdf]

Supporting information for "2D-3D integration of hexagonal boron nitride and a high-κ dielectric for ultrafast graphene-based electro-absorption modulators"

Hitesh Agarwal[1,*], Bernat Terrés[1,*,†], Lorenzo Orsini[1,2], Alberto Montanaro[3], Vito Sorianello[3], Marianna Pantouvaki[4], Kenji Watanabe[5], Takashi Taniguchi[5], Dries Van Thourhout[4], Marco Romagnoli[3], and Frank H. L. Koppens[1,6,‡]

[1]ICFO - Institut de Ciències Fotòniques, The Barcelona Institute of Science and Technology, 08860 Castelldefels (Barcelona), Spain
[2]Dipartimento di Fisica E. Fermi, Università di Pisa, Largo Bruno Pontecorvo 3, 56127 Pisa, Italy
[3]Consorzio Nazionale per le Telecomunicazioni (CNIT), Photonic Networks and Technologies National Laboratory, via Moruzzi 1, 56124 Pisa, Italy
[4]Photonics Research Group, Department of Information Technology, Ghent University-IMEC, Sint-Pietersnieuwstraat 41, Gent, 9000 Belgium
[5]National Institute for Materials Science, 1-1 Namiki, Tsukuba 305-0044, Japan
[6]ICREA - Institució Catalana de Recerça i Estudis Avancats, Barcelona, Spain
[*]These authors contributed equally
[†]bernat.terres@icfo.eu
[‡]frank.koppens@icfo.eu

December 17, 2020

Supplementary Note 1 Optical conductivity model

For the simulation of the modulators' optical transmission, we implemented the optical conductivity (σ) model of graphene based on Kubo's formula [1] [2].

$$\sigma = \sigma_{\text{intra}} + \sigma_{\text{inter}}, \tag{1}$$

where:

$$\sigma_{\text{intra}} = \frac{\sigma_{\text{o}}}{\pi} \frac{4}{\hbar\gamma - i\hbar\omega} \Big[E_{\text{F}} + 2k_{\text{B}}\text{T} \ln(1 + e^{-E_{\text{F}}/k_{\text{B}}\text{T}}) \Big], \tag{2}$$

$$\sigma_{\text{inter}} = \frac{\sigma_{\text{o}}}{2} \Big[tanh(\frac{\hbar\omega + 2E_{\text{F}}}{4k_{\text{B}}\text{T}}) + tanh(\frac{\hbar\omega - 2E_{\text{F}}}{4k_{\text{B}}\text{T}}) \Big] - i\frac{\sigma_{\text{o}}}{2\pi} ln\Big[\frac{(\hbar\omega + 2E_{\text{F}})^2}{(\hbar\omega - 2E_{\text{F}})^2 + (2k_{\text{B}}\text{T})^2} \Big], \tag{3}$$

with $\sigma_0 = q^2/(4\hbar)$ being the universal conductivity of graphene [3], ω the frequency, q the elementary charge, $\hbar$ reduced Planck's constant, k_{B} the Boltzmann constant, $k_{\text{B}}T$ the thermal energy, $\hbar\omega$ the photon energy, and γ the intra-band scattering rate, defined as:

$$\gamma = \frac{q\nu_{\text{F}}}{\hbar\mu\sqrt{\pi n}}, \tag{4}$$

where $\nu_{\text{F}} = 9.5 \text{ x } 10^7$ cm sec^{-1} is the Fermi velocity in graphene, n_{s} the free charge carrier density and μ the carrier density-dependent mobility. Both the carrier density-dependent mobility and the resulting intra-band scattering rate are extracted experimentally from a modulator device in Supplementary Note 2 (see red star points in Fig. S3). The Fermi energy (E_{F}) is defined as [4]:

$$E_{\text{F}} = sgn(n_{\text{s}})\hbar\nu_{\text{F}}\sqrt{\pi|n_{\text{s}}|}, \tag{5}$$

and the charge carrier density n_{s} [5]:

$$n_{\text{s}} = \frac{CV_{\text{BT}}}{qS}, \tag{6}$$

where V_{BT} is the voltage between the top and bottom graphene electrodes, S the surface of the capacitor and C the total capacitance. The total capacitance considers the top layer graphene quantum capacitance ($C_{\text{Q}}^{\text{top}}$), the parallel plate capacitance (C_{ox}) (refer to Supplementary Note 7) and the bottom layer graphene quantum capacitance ($C_{\text{Q}}^{\text{bottom}}$) [6]:

$$\frac{1}{C} = \Big(\frac{1}{C_{\text{Q}}^{\text{top}}} + \frac{1}{C_{\text{Q}}^{\text{bottom}}} + \frac{1}{C_{\text{ox}}} \Big), \tag{7}$$

where:

$$C_{\text{Q}}^{\text{bottom}} = C_{\text{Q}}^{\text{top}} = \frac{2qk_{\text{B}}T}{\pi(\hbar\nu_{\text{F}})^2} \log \Big[2\Big(1 + \cosh \frac{E_{\text{F}}}{k_{\text{B}}T}\Big)\Big]. \tag{8}$$

We determine the real part of the optical surface conductivity (σ/σ_0) as a function of the charge carrier density (n) and the incoming photon energy ($\hbar\omega$) following the model described above and the graphene electronic mobility derived in Supplementary Note 2 (see Fig. S1a) . For the DataCom wavelength λ = 1550 nm ($\hbar\omega$ = 0.8 eV, black dashed line in Fig. S1a), the real part of σ/σ_0 (i.e. the absorption term) decreases significantly at E_{F} > 0.4 eV, i.e. when the device reaches Pauli blocking regime due to the lack of inter-band transitions (E_{F} > $\hbar\omega/2$), which results in a sharp transition (at zero kelvin and for very high mobility of graphene) (black trace in Fig. S1b). The onset of the Pauli blocking regime is thus defined as $\hbar\omega/2$ = 0.4eV at 0K. At room-temperature (T=300K), this transition broadens significantly (red trace in Fig. S1b). We thus define the Pauli blocking onset as 0.5 eV at room-temperature

(see vertical dashed line in Fig. S1b and orange-shaded region in Fig. 3b and c of the main text or in Figs. S11 and S12). Beyond this Fermi energy onset, ∼ 98% of the incoming light is blocked (for high quality devices) and we therefore consider to be in Pauli blocking regime.

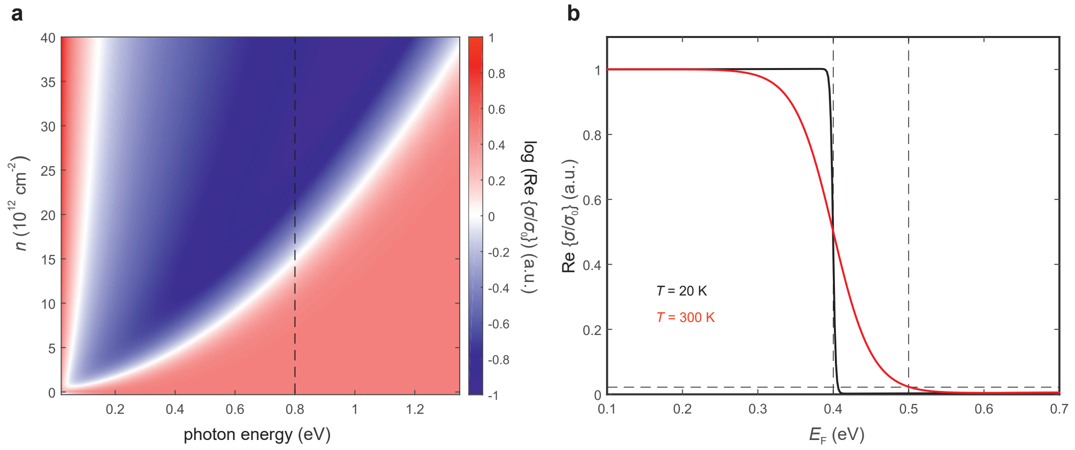

Supplementary Figure S1. Optical absorption in graphene. a) Graphene absorption as a function of the laser photon energy ($\hbar\omega$) and the graphene charge carrier density (n). This map is calculated considering T=300K and the graphene electronic mobility derived in Supplementary Note 2. The vertical black dashed line indicates the photon energy for an excitation laser of λ=1550 nm. **b**) Graphene absorption as a function of the graphene Fermi energy E_F for two distinct temperatures. At room-temperature (T=300K) and for E_F>0.5eV ∼ 98% of the incoming light is blocked.

Supplementary Note 2 Mobility extraction

All modulators in the main manuscript have been fabricated using the hot-pickup technique [7] [8]. To characterize the fabrication method, we first analyze by Raman spectroscopy measurements the quality of a resulting hBN-graphene-hBN stack (Fig. S2a). The full-width-half-maximum of the 2D Raman peak (Γ_{2D}) is uniform across the whole flake surface (see inset of Fig. S2b), with a Γ_{2D} mean value of $\sim$ 18.1 cm^{-1} (Fig. S2b). This low value of Γ_{2D} is characteristic of high-quality single-layer graphene [9]. Moreover, the scattering plot indicates low levels of doping and moderate strain in the sample (Fig. S2c).

To confirm the high quality of the hBN-graphene-hBN stack, we fabricated a Hall bar device (inset of Fig. S2d) out of the analyzed stack (Fig. S2a, b and c). We extract a residual doping of n^*=5.94 x 10^{10} cm^{-2} and a mobility of 103,000 and 96,000 cm^{2}V^{-1}s^{-1} for the electron (red curve) and hole (blue curve) doping, respectively (Fig. S2d). The model used to extract these electrical parameters considers a constant mobility as a function of the charge carrier density ref. [10].

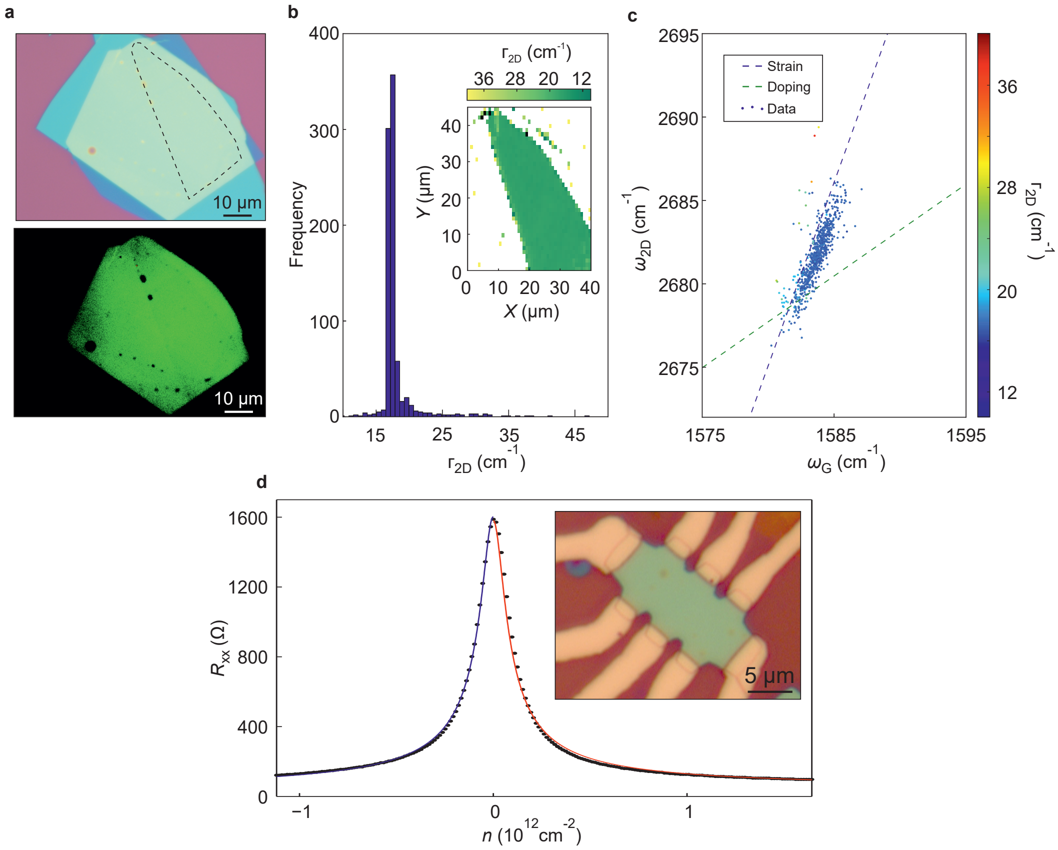

Supplementary Figure S2. Hall Bar reference device. **a**) Optical image of a hBN-graphene-hBN heterostructure fabricated using the hot pick-up technique [7] [8]. The graphene flake (black dashed line in top panel) is optically visible when viewed with enhanced contrast (bottom panel). **b**) Histogram plot of the Raman scan (see inset) showing the full-width-half-maximum of the graphene 2D Raman peak (Γ_{2D}). **c**) Scattering plot of the 2D-peak versus the G-peak frequency (ω_{2D} and ω_G, respectively). The color bar represents the Γ_{2D} of the recorded spectrum (inset of panel **b**). **d**) Four-probe longitudinal resistance (R_{xx}) (black data points) as a function of the charge carrier density (n) of the measured Hall bar device (see inset). The red and blue traces indicate the conductance model for the electron and hole sides, respectively (ref. [10]).

To obtain an accurate measure of the modulator's mobility, we extract the mobility from a relevant device, i.e. a device fabricated on top of an optical waveguide. For that, we double-contacted the top and bottom graphene electrodes of a graphene modulator fabricated on top of an optical waveguide (see insets in Fig. S3a). The resistance as a function of the applied voltage V_{BT} is represented by black data points and the numerical fitting to the conductance model (ref. [10]) by blue and red solid traces (right inset of Fig. S3a). To compare the electronic mobility of this device with literature (refer to the main text), we extract the carrier-independent mobility using the conductance model from ref. [10]. We find a carrier-independent mobility as high as 30,000 cm^{2}V^{-1}s^{-1} at room temperature for the holes side (red trace in right inset of Fig. S3a). The graphene appears slightly doped, with a charge-neutrality point located at V_0 = -0.91 V (dashed vertical line in Fig. S3a), corresponding to a Fermi energy of 0.172 eV.

However, to accurately simulate the modulator's transmission, it is mandatory to extract the mobility as a function of the charge carrier density. The commonly-used framework for extracting the mobility is the Drude conductance: $\sigma = \mu e n$, where μ is the charge carrier-

dependent Drude mobility and n is the charge carrier density.

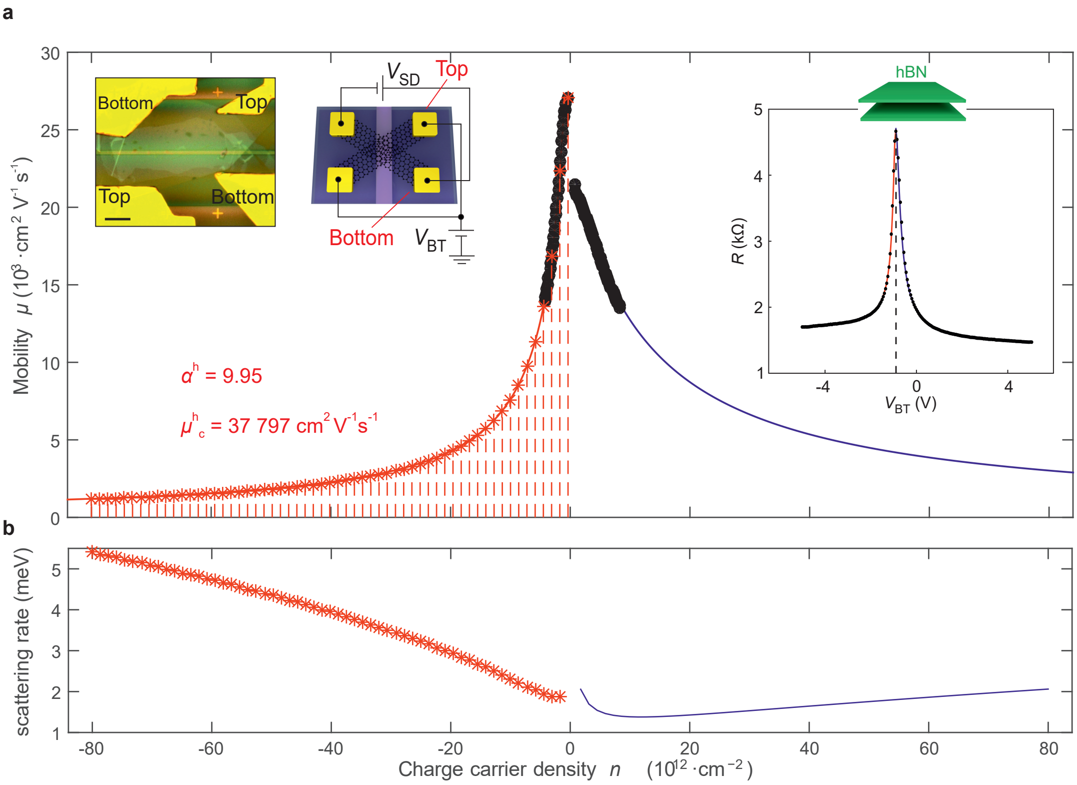

Supplementary Figure S3. Charge carrier density dependent Mobility and scattering rate of the hBN based modulator. a) Mobility (black data points) as a function of the charge carrier density for the hBN-encapsulated graphene modulator shown on the left inset (scale bar: 10 µm). The mobility is extracted from the resistance curve shown in the right inset, where we show the resistance R of the bottom graphene electrode as a function of the voltage between the top and bottom graphene electrodes (V_{BT}). A source-drain voltage $V_{\mathrm{SD}} = 10$ mV is applied between the two terminals of the bottom graphene (see the electrical connections in the left inset). The blue and red solid lines in the right inset are fits [10] to the data (black data points). Measurements were taken at room temperature. **b**) Scattering rate for the holes (red) and electrons (blue) sides, derived from the mobility in panel **a** (see details in Supplementary Note 1).

Fig. S3a shows the calculated μ values (black data points) for the electrons and holes based on the measurements shown in the right inset of Fig. S3a. To extrapolate the mobility towards high charge carrier densities, we fit the extracted mobility (back dots in Fig. S3a) with the following model [11]:

$$\mu^{-1} = \mu_{\mathrm{c}}^{-1} + \mu_{\mathrm{sr}}^{-1}, \tag{9}$$

where μ_{c} represents the mobility limited by Coulomb scattering and μ_{sr} the mobility limited by short-range scattering events [11]. This model is valid at room-temperature, where the scattering mechanism is dominated by impurities [11]. The first mobility term (μ_{c}) is constant as a function of the charge carrier density n, while μ_{sr} is inversely proportional to n ($\mu_{\mathrm{sr}} = 2\pi\alpha/\hbar n$) [11]. The result of the fitting is shown in Fig. S3a by the red and blue solid curves, representing the hole and electron regimes, respectively The extracted μ_{c} and α terms for the holes side are indicated in red in Fig. S3a. For the electro-optical simulations, we use the lowest mobility values, which in our case, correspond to the holes side (see red star points in Fig. S3a).

Supplementary Note 3 FDTD simulations

To confirm the experimental transmission curves (Fig. 1e and Fig. 3b and c of the main text), we simulated our double-layer (DL) graphene modulator devices using the optical conductivity model introduced in Supplementary Note 1 and a commercial FDTD software (Lumerical). The values of mobility and intrinsic doping used in the simulations are determined in Supplementary Note 2 and the relative permittivity of the hBN, HfO_2 and hBN-HfO_2-hBN extracted in Supplementary Note 5 and Supplementary Note 10. All devices have been simulated with the same geometry (thicknesses, widths and lengths) as the measured ones. In the FDTD software, we treat graphene as an equivalent 3D layer of thickness h_G=0.34nm [12], with the out-of-plane dielectric constant of graphite and in-plane dielectric constant given by [13]:

$$\epsilon_G(\omega) = 1 + \frac{i\sigma(\omega)}{\omega\,\epsilon_0\,h_G} \tag{10}$$

where ϵ_0 is the vacuum permittivity and $\sigma(\omega)$ is defined in Supplementary Note 1. The cross-section of the silicon (Si) waveguide used in Fig. 1e can be seen in Fig. S4a and the obtained mode profile is shown in Fig. S4b, with the modulator structure on top.

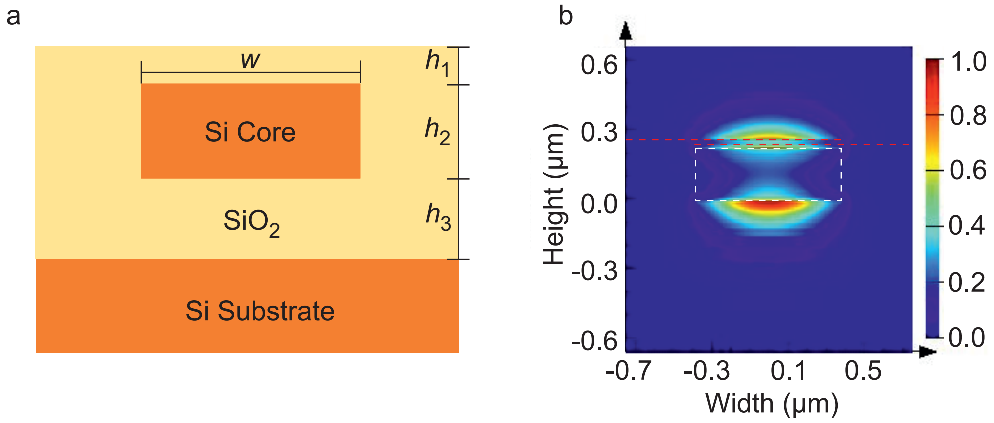

Supplementary Figure S4. Cross-section and optical mode profile of the SOI waveguide. **a**) Cross-section of the SOI waveguide used for the device in Fig. 1e. The dimensions are as follows: h_1=10nm (SiO_2 cladding), h_2=220nm, h_3=2µm, and w=750nm (optimized for the TM mode [14]). **b**) Optical mode profile of the device in Fig. 1e. The device is based on a DL graphene (red dashed lines) modulator separated by a hBN-HfO_2-hBN dielectric on top of a 750nm-wide SOI waveguide (white dashed rectangle, see panel **a**). The waveguide is optimized for 1550nm wavelength and is designed to carry a single TM mode. The color bar represents the intensity of the electric field $|\vec{E}^2|$ in arbitrary units.

Supplementary Note 4 Transfer-length-method (TLM) measurements of Cr-Pd-Au 1D edge contacts on hBN-encapsulated graphene

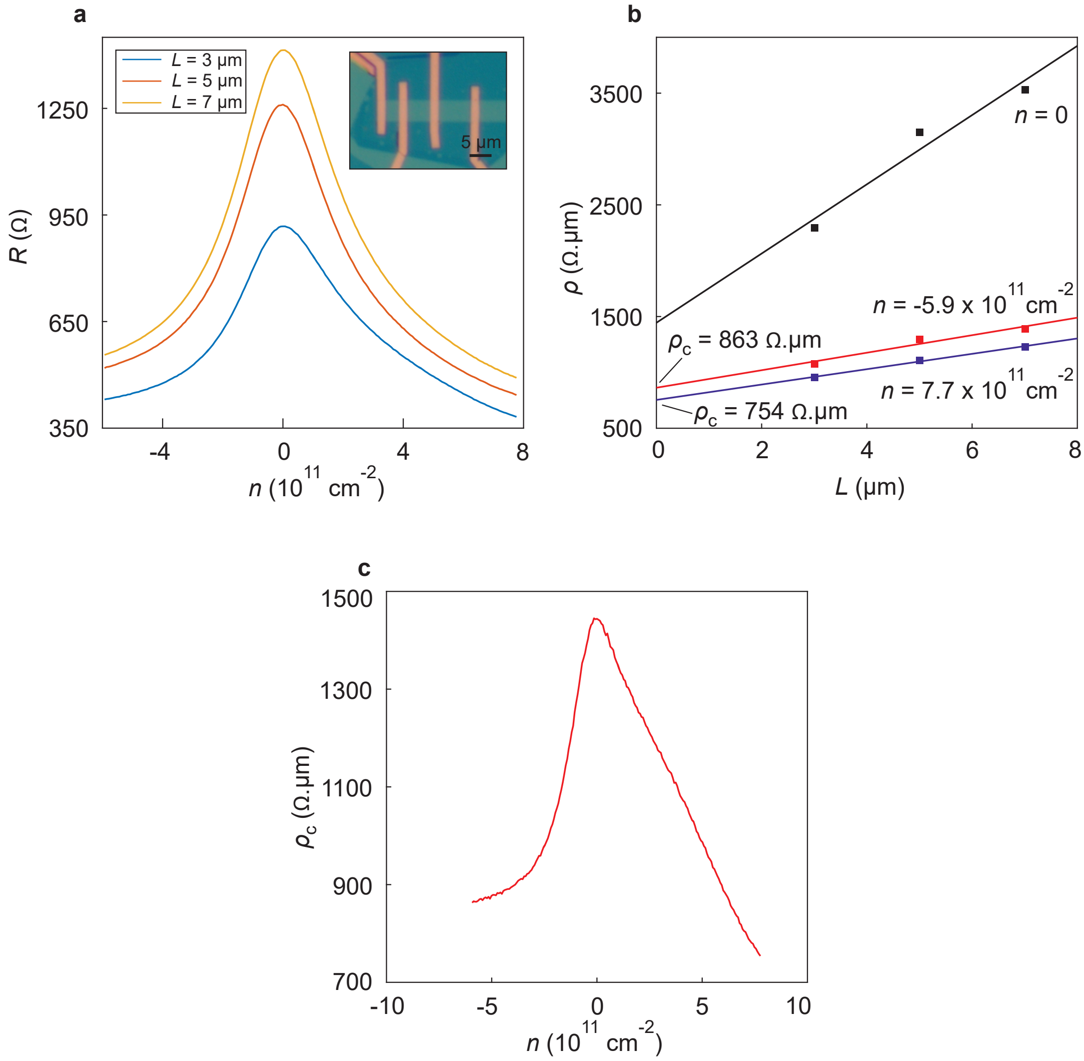

Supplementary Figure S5. Extraction of the contact resistivity. a) Two-probe resistance (R) as a function of charge carrier density (n) for different channel length (L). Temperature is 300 K and the source-drain voltage applied is 1 mV. Inset shows an optical image of the TLM device with edge-contacts. **b**) Two-probe resistivity (ρ) versus L at fixed n. Contact resistivity (ρ_c) is calculated from the least-squares fitting (line) for fixed n [15]. **c**) ρ_c (calculated as shown in panel **b**) as a function of n. ρ_c does not saturate here indicating the actual value of ρ_c might be much lower at higher n.

Supplementary Note 5 hBN, HfO_2 and hBN-HfO_2-hBN characterization

To characterize the hBN, HfO_2 and hBN-HfO_2-hBN dielectrics, we fabricated metal-insulator-metal (MIM) capacitors with circular metal electrodes on the top and below the dielectric under study (see insets of Fig. S6). To fabricate the MIM devices, we exfoliated the hBN flakes and dry transferred [7] [8] onto the bottom metal electrodes. The HfO_2 has been grown by atomic layer deposition (ALD) method in a Savannah G1 system from Cambridge Nanotech. The best results were achieved using the recipe E (see table 1). All the HfO_2-based MIM devices analyzed in this section have been fabricated with this ALD recipe E. We measured the leakage current (I) as a function of the applied electric field (E) (Fig. S6a and c) and defined the breakdown field (E_{BD}) as the electric field at a leakage current I=10nA (see red dashed lines in Fig. S6a and c). As observed in Fig. S6b, d, the extracted E_{BD} values (black data points) do not show any significant dependence on the area of the electrodes (A). We obtain an averaged breakdown field of 7.6 MV cm^{-1} and 5.5 MV cm^{-1} for the hBN and HfO_2 dielectrics, respectively (Fig. S6b, d).

To determine the dielectric constant (ϵ_{r}), we plotted the capacitance as a function of area and extracted the relative permittivity ϵ_{r} using a linear fit to the data [16]. For the hBN dielectric, we plot the capacitance scaled by the hBN thickness (Fig. S6b) to account for the different hBN thickness of the devices. We extract a relative permittivity of 3.8 (11.93) for the hBN (HfO_2) dielectric (see red dashed linear fits in Fig. S6b and d). Our results are in good agreement with literature [17] [18] [19].

To characterize the breakdown field of the hBN-HfO_2-hBN dielectric used in the modulators of Fig. 1 and Fig. 3c of the main text, we fabricated three capacitors with a hBN-HfO_2 dielectric. We note that the results of the hBN-HfO_2 MIM capacitor can be directly translated to a hBN-HfO_2-hBN dielectric of the same HfO_2 and total hBN thicknesses. All devices (Fig. S7a) have the same 10nm-thick HfO_2 layer and the total hBN thickness indicated in the inset of Fig. S7a. As expected the breakdown electric field values scale with the hBN thickness (Fig. S7b). We evaluated a breakdown field of $E_{\mathrm{BD}} \approx 8.9\,\mathrm{MV\ cm^{-1}}$ (see black dashed lines in Fig. S7b) for the modulator device of the main text (Fig. 1e), i.e. for a 10 nm-thick HfO_2 and a 17 nm total hBN thickness.

To characterize the quality of the HfO_2 grown over the hBN layer, we analyze the roughness (Rq) extracted by atomic force microscopy (AFM) measurements (see Fig. S8). The roughness indicates how uniform the covering of the HfO_2 layer is, which is crucial for the performance of the dielectric. Furthermore, to confirm that the HfO_2 is suited for Pauli blocking operation (i.e. can withstand Fermi energy > 0.5 eV for $\lambda = 1550$ nm), we defined the operation field E_{o} for each HfO_2 growth recipe and compared it with their respective E_{BD} [20]. The comparison of all these parameters (Rq, ϵ_{r}, E_{BD} and E_{o}) for the different growth recipes, is summarized in table 1. As observed, the best results are achieved using recipe E (see table 1).

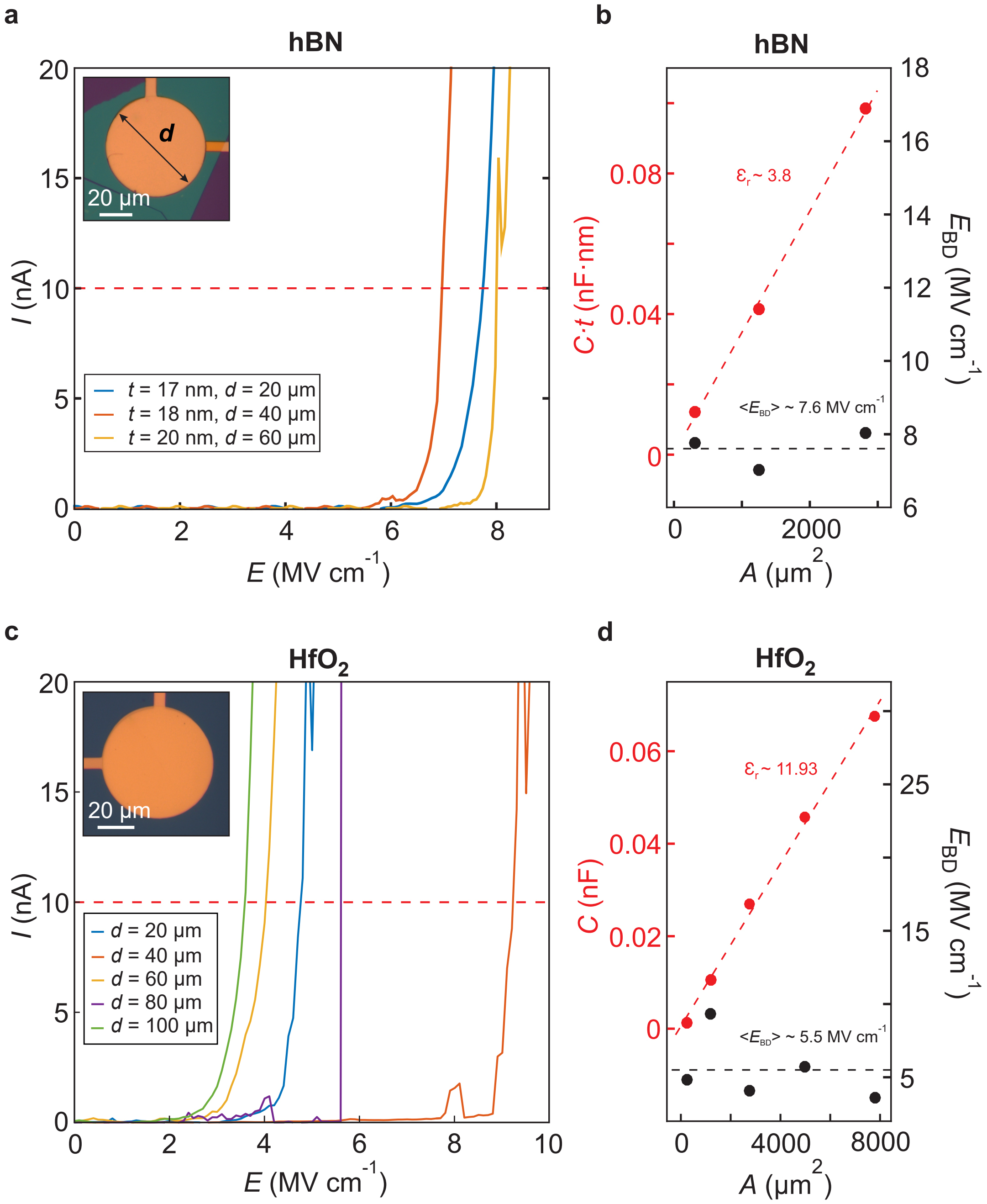

Supplementary Figure S6. hBN and HfO_2 dielectric characterization. **a**) and **c**) Leakage current (I) versus electric field (E) between the top and bottom circular metal electrodes (see inset) for hBN (panel **a**) and HfO_2 (panel **c**) dielectrics. The dashed red line at $I = 10$ nA represents the current threshold defining the breakdown field E_{BD} (see panels **b** and **d**), and d corresponds to the diameter of metal electrodes (see insets). **b**) and **d**) Experimentally determined capacitance normalized by the dielectric thickness $C.t$ (panel **b**), and capacitance C (panel **d**) (red-colored left axis) as a function of the area $A = \pi(d/2)^2$. The dashed red line are the linear fits used to extract ϵ_{r}. The black-colored right axis indicates the breakdown field E_{BD} and the black-dashed line the average value of E_{BD}.

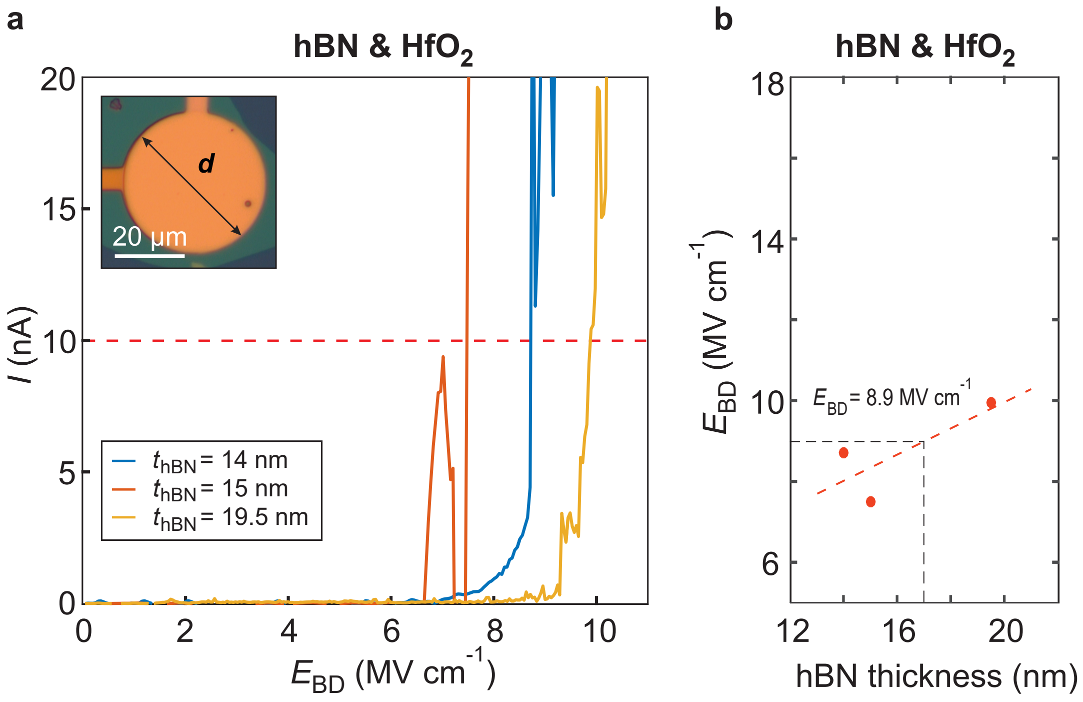

Supplementary Figure S7. hBN-HfO_2 dielectric characterization. **a**) Leakage current (I) versus electric field (E) between the top and bottom circular metal electrodes (see inset) for hBN-HfO_2 dielectrics. The HfO_2 thickness is 10 nm in all three cases. The hBN thicknesses (t_{hBN}) are indicated in the inset. The dashed red line at $I = 10$ nA represents the current threshold defining the breakdown field E_{BD} (see panel **b**) and d is the metal electrode diameter. **b**) Breakdown field E_{BD} as a function of the hBN thickness. The red dashed line is a linear fit to the data and the noted breakdown field value of $E_{\mathrm{BD}} = 8.9\,\mathrm{MV\ cm^{-1}}$ is the expected E_{BD} for the modulator device in Fig. 1e of the main text (see Supplementary Note 10).

Supplementary Table 1. Optimization growth of HfO_2.

Recipe	T(°C)	Purge time (s)	Seed layer	t (nm)	Rq (nm)	ϵ_r	E_o (MV cm^{-1})	E_{BD} (MV cm^{-1})
A	250	5	-	30	-	12.77	2.8	-
B	250	5	-	10	3.33	10.99	3.28	2.32
C	200	20	-	10	0.52	5.4	6.66	5.71
D	250	5	2nm Si	12	2.98	6.74	5.34	6.76
E	250	5	2nm SiO_2	12	0.26	11.93	3.02	5.5

- The purge time applies after the treatment with H_2O and Tetrakis(dimethylamido)-hafnium (TDMAH) and t represents the thickness of HfO_2 and the seed layer.
- The root mean square roughness (Rq) is analyzed in a 0.4 μm^2 area for HfO_2 grown over hBN.
- E_o (Operation field) is the field required to withstand Fermi energy larger than 0.5 eV (Pauli blocking regime with $\lambda = 1550$ nm) [20].

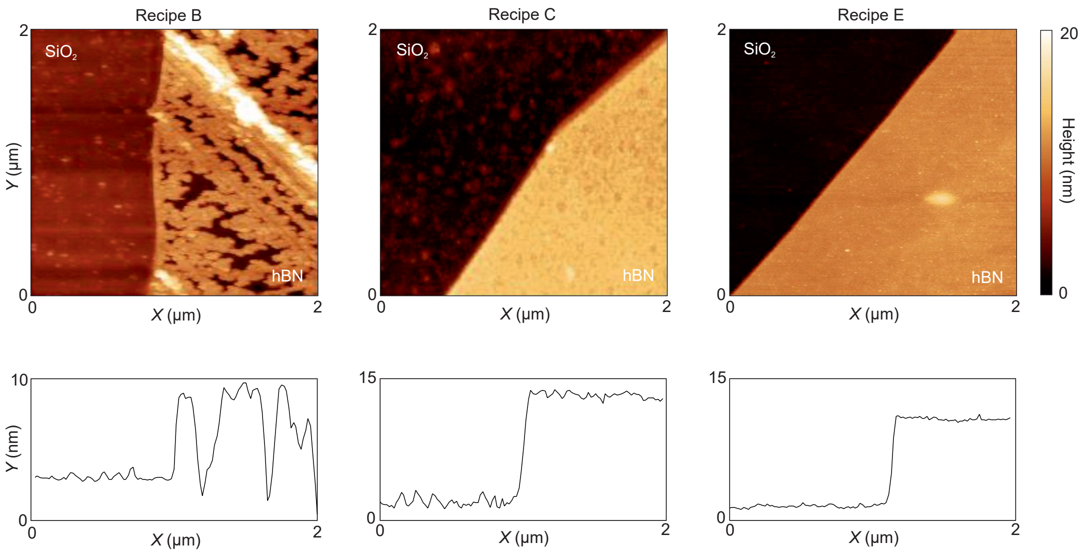

Supplementary Figure S8. Topography characterization of the HfO_2 growth recipes. Atomic force microscopy (AFM) topography scans of 10 nm thick HfO_2 grown over hBN and SiO_2. The root mean square roughness values (Rq) on hBN (analyzed for a 0.4 μm^2 area) for recipe B, C and D are 3.33, 0.52 and 0.26nm respectively. For more details (growth parameters) refer to table 1

Supplementary Note 6 Raman characterization

To quantify the degradation in graphene quality due to HfO_2 growth, we compared the Raman spectra of hBN based EA modulators (see Fig. S9a and b) and hBN-HfO_2-hBN based EA modulators (see Fig. S9c and d). Both devices show very low doping, strain and comparable histograms (see Fig. S9b and d), with mean full-width-half maximum of the graphene 2D Raman peak (Γ_{2D}) $\sim$ 24.1 and 18.4 cm^{-1}, respectively, characteristic of high-quality single-layer graphene [9]. This analysis suggested the quality of DL modulator is independent of the dielectric between two graphene electrodes and hence, HfO_2 layer does not seem to degrade the quality of graphene.

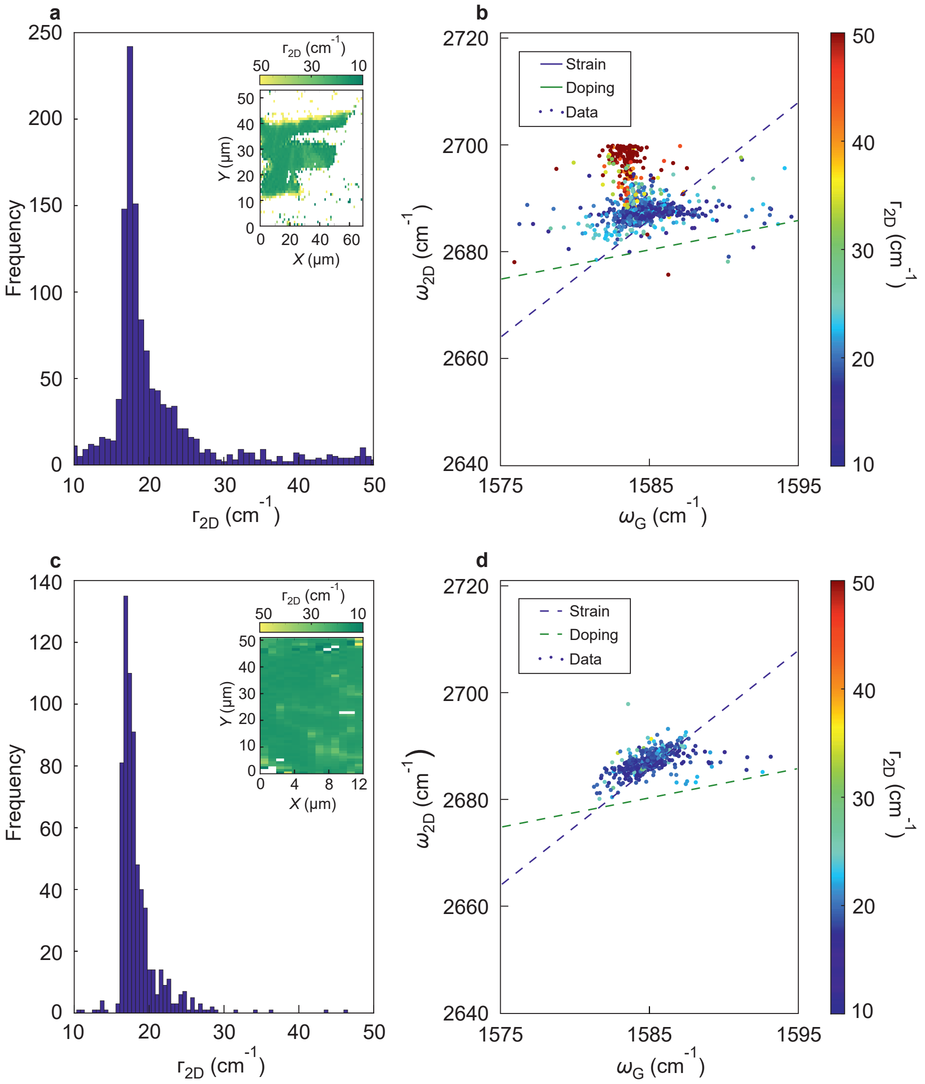

Supplementary Figure S9. Raman characterization of the hBN and hBN-HfO_2-hBN based modulators. a) and **c**) Histogram plot of the Raman scan (see inset) showing the full-width-half-maximum of the graphene 2D Raman peak (Γ_{2D}) of hBN based EA modulator (panel **a**) and hBN-HfO_2-hBN based EA modulator (panel **c**) over the waveguide. **b**) and **d**) Scattering plot of the 2D-peak versus the G-peak frequency (ω_{2D} and ω_G, respectively) of hBN based EA modulator (panel **b**) and hBN-HfO_2-hBN based EA modulator (panel **d**), where the color bar represents the Γ_{2D} of the recorded spectrum (inset of the panel **a** and **c**).

To check the quality of our DL graphene EA modulator presented in the main text (see Fig. 1), we measured the Raman spectra of the full modulator (hBN-graphene-hBN-HfO_2-hBN-graphene-hBN) over the silicon waveguide (see Fig. S10a). The analyzed data shows scattering plot and histogram, with average values of the 2D-peak full-width-half-maxima Γ_{2D} = 27.9 cm^{-1}, low doping and moderate levels of strain, characteristic of a high quality graphene (see Fig. S10b) [9]. The high value of Γ_{2D} in this device is due to the very thin bottom hBN (5 nm) and highly rough SiO_2 substrate.

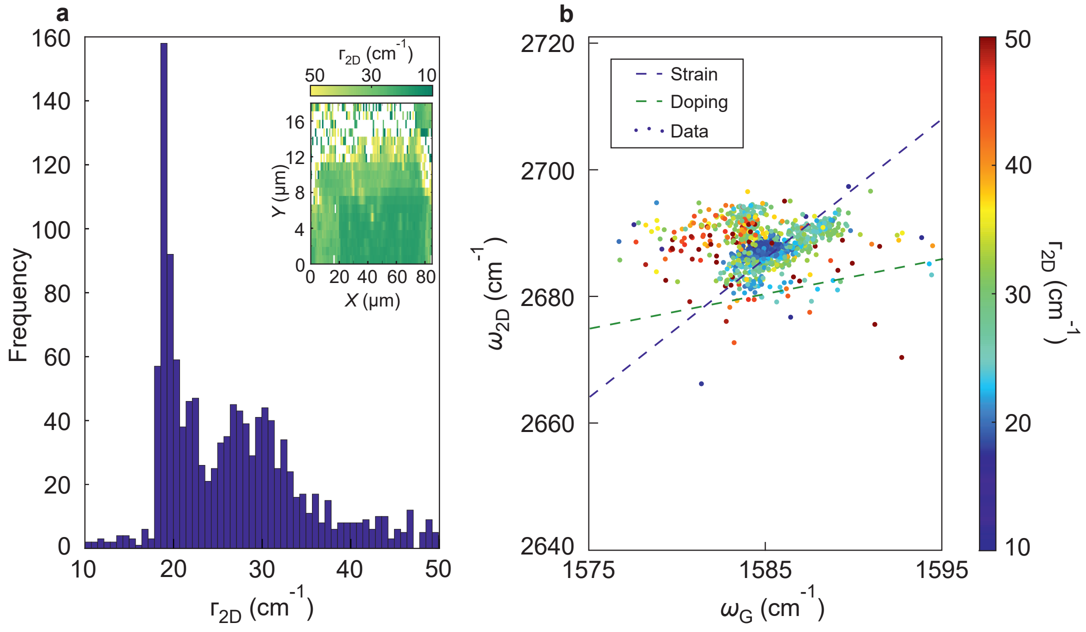

Supplementary Figure S10. Raman characterization of modulator device in Fig. 1 of the main text. **a**) Histogram plot of the Raman scan (see inset) showing the full-width-half-maximum of the graphene 2D Raman peak (Γ_{2D}) of the EA modulator ((hBN-graphene-hBN-HfO_2-hBN-graphene-hBN) over the silicon waveguide presented in the main text (see Fig. 1). **b**) Scattering plot of the 2D-peak versus the G-peak frequency (ω_{2D} and ω_G, respectively) of the same device, where the color bar represents the Γ_{2D} of the recorded spectrum (inset of the panel **a**).

Supplementary Note 7 Relation between E_F and V_{BT}

To compare the breakdown of the different dielectrics, we represent the transmission curve of the modulators as a function of the Fermi energy E_F at the graphene electrodes. Representing the transmission as a function of E_F instead of V_{BT} allows us to rule-out the different thicknesses and relative permittivity of the compared dielectrics. Neglecting the contribution of the quantum capacitance, we define the Fermi energy as:

$$E_F = sgn(n_s)\hbar\nu_F\sqrt{\pi|n_s|}, \tag{11}$$

where $\hbar$ and ν_F are defined in Supplementary Note 1, and the charge carrier density at the graphene electrodes n_s relates to V_{BT} as follows:

$$V_{BT} = \frac{qSn_s}{C_{ox}}. \tag{12}$$

S is the area of the capacitor (defined by the overlap area between the top and bottom graphene electrodes), q the elementary charge and C_{ox} the total capacitance, defined for modulators with a hBN-hBN dielectric as:

$$\frac{1}{C_{ox}} = \frac{1}{C_{hBN_1}} + \frac{1}{C_{hBN_2}} = \frac{d_{hBN_1} + d_{hBN_2}}{S\epsilon_{hBN}}, \tag{13}$$

and for modulators with a hBN-HfO_2-hBN dielectric as:

$$\frac{1}{C_{ox}} = \frac{1}{C_{hBN_1}} + \frac{1}{C_{HfO_2}} + \frac{1}{C_{hBN_2}} = \frac{d_{hBN_1} + d_{hBN_2}}{S\epsilon_{hBN}} + \frac{d_{HfO_2}}{S\epsilon_{HfO_2}}. \tag{14}$$

d_{hBN_1}, d_{hBN_2} and d_{HfO_2} are the thicknesses of the hBN layers and the HfO_2 oxide in between the top and bottom graphene electrodes. The relative permittivity ϵ_{hBN}=3.8 is experimentally obtained (see Supplementary Note 5). The relative permittivity for hafnia ϵ_{HfO_2}=9.5 is extracted from the comparison between simulation and data (see Supplementary Note 10). After the conversion from V_{BT} to E_F, we plot the transmission traces of Fig. 1e and 3b in the main text as a function of both E_F and V_{BT}. See Fig. S11 for an individual representation of the transmission traces of Fig. 3b.

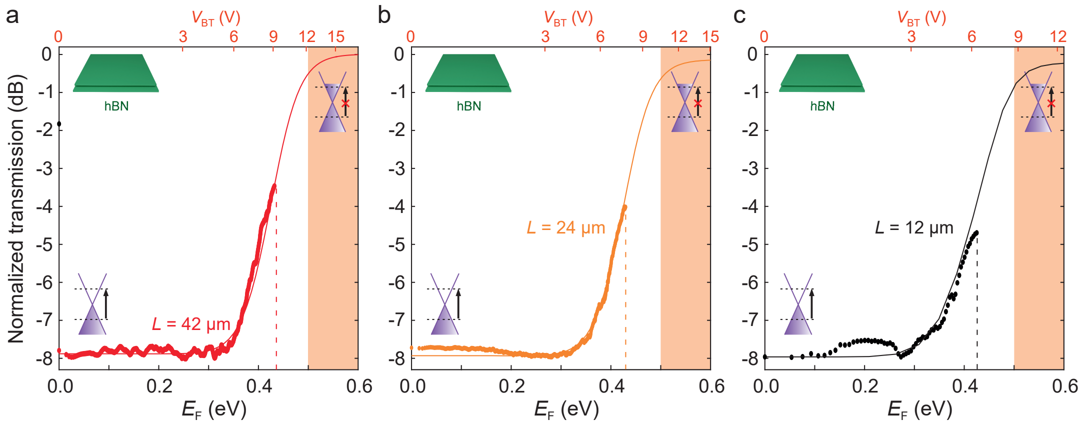

Supplementary Figure S11. Transmission curves of hBN-based modulators. Individual transmission curves of the hBN-based devices shown in Fig. 3b of the main text plotted with duals axis E_F and V_{BT}.

Supplementary Note 8   Dielectric breakdown

To understand the effect of the dielectric breakdown on the transmission, we intentionally swept the V_{BT} beyond the breakdown voltage of the dielectric. As observed in Fig. S12a, where we show the complete transmission trace of the 24 µm-long device from Fig. 3b, the transmission drops suddenly beyond the dielectric breakdown, making the device inoperative.

To prevent damaging the modulators, we continuously monitored the leakage current through the devices (Fig. S12c). As observed in in Fig. S12b and c, the hBN dielectric broke before reaching the Pauli blocking regime (see grey- and orange-shaded regions in Fig. S12b and c). This is characterized by a sudden increase in current between the top and bottom graphene electrodes (leakage current) (Fig. S12c). Monitoring the leakage current allows us to determine the breakdown voltage of the different dielectrics.

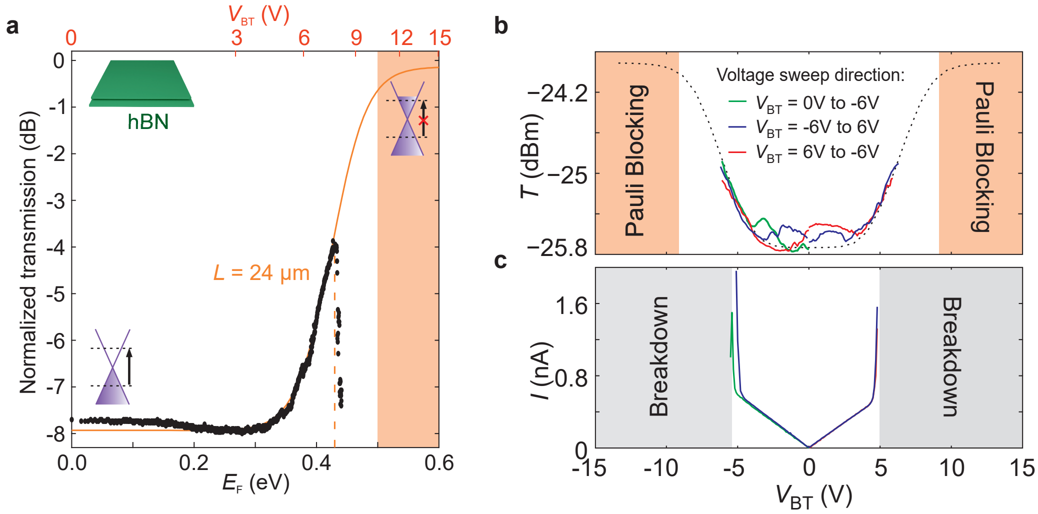

Supplementary Figure S12. Characteristic dielectric breakdown of a hBN-based modulator. **a**) Transmission curve for the 24 µm-long device in Fig. 3b of the main text, showing the effect of the dielectric breakdown on the transmission. The black dots are data points and the solid curve the simulated transmission. **b**) Transmission (T) as a function of the voltage applied between the top and bottom graphene electrodes (V_{BT}). **d**) Leakage current (I) as a function of V_{BT}.

Supplementary Note 9 Hysteresis

Additional to the hBN-HfO_2-hBN based modulators presented in Fig. 1 and Fig. 3c of the main text, we characterize the hysteresis of a third hBN-HfO_2-hBN based modulator (see Fig. S13) to further support the reduced hysteresis and the symmetric operation enabled by this 2D-3D dielectric combination. This third hBN-HfO_2-hBN device shows the lowest modulation efficiency, mainly due to its shorter length (L=15 µm).

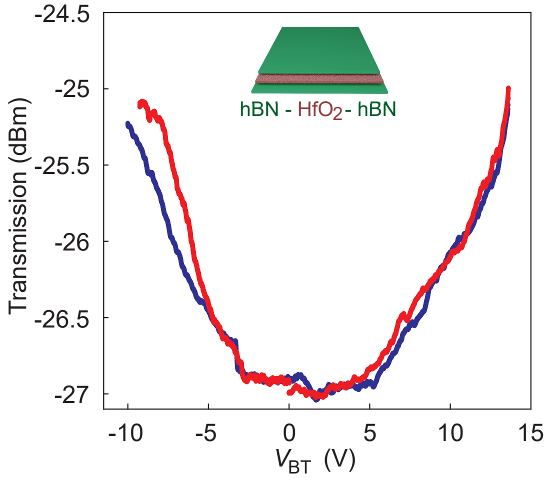

Supplementary Figure S13. Hysteresis of hBN-HfO_2-hBN based modulators. Transmission curves as a function of V_{BT} for a DL graphene modulator with a hBN-HfO_2-hBN dielectric and a length $L = 15\mu$m. The forward and backward voltage sweeps (blue and red, respectively) show no major hysteresis.

Supplementary Note 10 Evaluation of the Insertion Loss

To evaluate the Insertion Loss (IL) of our devices, we compare the measured and the simulated transmission curves (see Figs. 3b and c of the main manuscript and Figs. S14 and S15). Fig. S14 shows the simulated transmission (dashed green trace) overlapping the experimental transmission curve of Fig. 3c (black data points). We emphasize that the simulated devices have the same geometry as the measured ones, the optical conductivity model implemented in the simulations includes both the intra- and the inter-band contributions (Supplementary Note 1) and the charge carrier-dependent mobility has been experimentally extracted from a relevant modulator device (Supplementary Note 2).

To highlight the agreement between simulations and data, we also plot in Fig. S14a the simulation curves from two equal devices but with slightly modified lengths ($L = 54\,\mu$m and 34 µm for the dashed red and blue traces, respectively). Neither the slope nor the saturation region (Pauli blocking at high V_{BT}) of these shorter and longer simulated devices (blue and red dashed traces in Fig. S14a) match the measured transmission (black dots in Fig. S14a), confirming the good agreement between simulations and measurements.

To evaluate the IL of our devices, we first introduce the intrinsic IL. As widely accepted in literature, the intrinsic IL of a graphene modulator is solely determined by the graphene quality, i.e. the electronic mobility (refer to Fig. 2d and Fig. 3c, d of [21], Fig. 2b and Fig. 4b of [22], Fig. 3b, d of [14] and Fig. 6a of [20]). In our case, the experimentally extracted carrier-dependent mobility (Fig. S3a) translates into a record low intrinsic IL$\approx 5.52 \cdot 10^{-3}$ dB

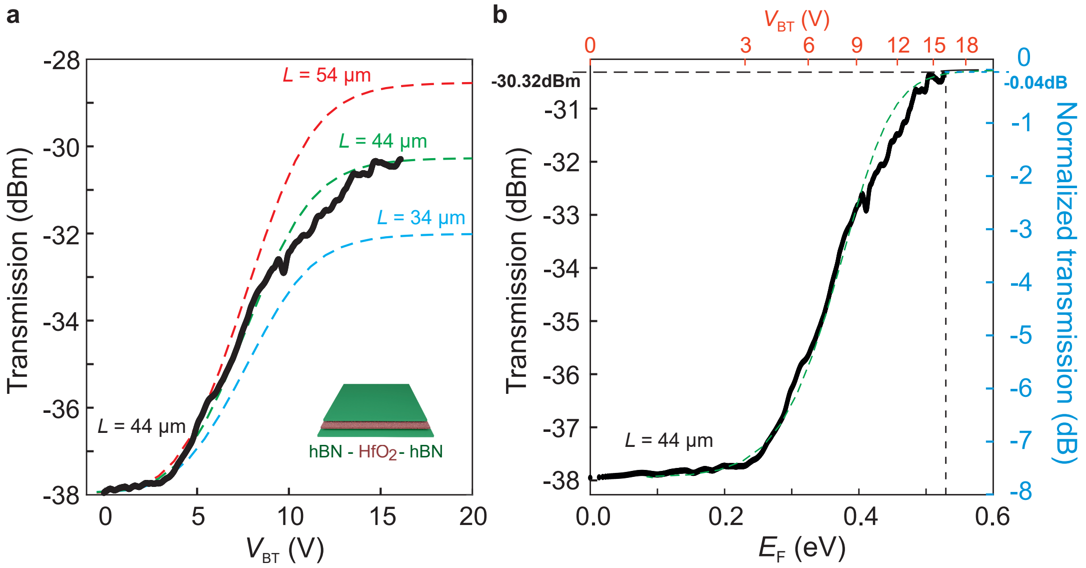

Supplementary Figure S14. Comparison with simulations for the device in Fig. 3c of the main text. a) Measured transmission (black data points) as a function of V_{BT} for the modulator device in Fig. 3c. The incoming power was set to $P_{\mathrm{in}} = 0$ dBm. The simulated transmission is shown by the dashed green trace. The red and blue dashed traces correspond to the simulated transmission of a $L = 54\,\mu$m and $L = 34\,\mu$m long devices, respectively. **b**) Transmission (black data points) shown in a) as a function of E_{F}. The V_{BT}-axis is also indicated (in red). The measured transmission (black left axis) includes the fiber-to-fiber losses of a pristine waveguide (16 dB) and the fabrication impurities deposited along the waveguide (evaluated as 30.32 dB - 16 dB = 14.32 dB). The normalized transmission (blue right axis) only considers the losses of the modulator device itself and therefore reflects the true performance of the device.

at 48V (see Fig. S15c). In practice, however, the effective IL is limited by how far the V_{BT} voltage can be increased. Meaning that the breakdown of the dielectric determines whether the intrinsic IL (dictated by the quality of graphene) can be achieved.

The IL of the hBN-HfO_2-hBN device in Fig. 3c is IL$\approx$ 0.04 dB (marked in blue in Fig. S14b). In that case the maximum applied $V_{\mathrm{BT}} \approx 16\,\mathrm{V}$ (or alternatively the maximum $E_{\mathrm{F}}^{\mathrm{max}} = 0.54\,\mathrm{eV}$, see vertical black dashed line in Fig. S14b) has been pushed up to the Pauli blocking regime without breaking the dielectric, i.e. the applied $V_{\mathrm{BT}} \approx 16\,\mathrm{V}$ lies below the breakdown limit of the hBN-HfO_2-hBN (Supplementary Note 5). This IL$\approx$0.04 dB exclude any fiber-to-fiber losses from the waveguide or any polymer residues deposited along the waveguide during the fabrication processes, thus reflecting the capabilities of the graphene modulator itself. The measured IL$\approx$30.32 dB (marked in black in Fig. S14b) is higher than the IL$\approx$0.04 dB since it includes the fiber-to-fiber losses of the waveguide (evaluated from a pristine waveguide at 16 dB) and the polymer residues deposited along the waveguide during the fabrication processes (evaluated as 30.32 dB - 16 dB =14.32 dB). This value (14.32 dB) is in agreement with the average losses ($\sim$ 14 dB) measured in four device-free (without graphene) optical waveguides that followed the same fabrication steps.

For the evaluation of the insertion loss of the device in the Fig. 1e, we repeat the same comparison between the simulated and the measured transmission curves. Fig. S15 shows the

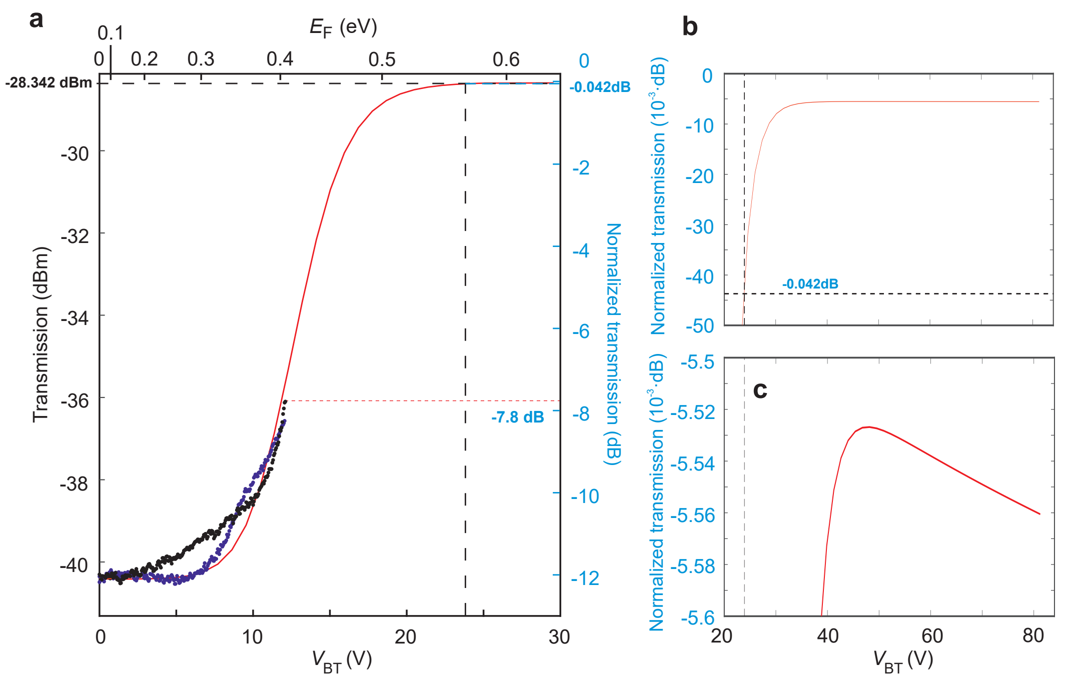

Supplementary Figure S15. Comparison with simulations for the device in Fig. 1e of the main text. a) Transmission curves as a function of V_{BT} and E_{F} for the modulator with a hBN-HfO_2-hBN dielectric of Fig. 1e. The forward (black) and backward (blue) voltage sweeps (also shown in Fig. 1e) overlap the simulation trace (in red). We marked in black the IL including the losses from fabrication residues along the waveguide IL$\approx$ 28.342 dB and in blue the measured IL$\approx$ 7.8 dB and the potential IL$\approx$ 0.042 dB. The vertical black dashed line defines the maximum V_{BT} voltage the dielectric is expected to withstand. **b**) Zoom-in at high V_{BT} voltages showing the crossing of the simulated transmission (red curve) and the maximum V_{BT} allowed by the dielectric (vertical dashed line). **c**) Zoom-in in the Pauli blocking regime showing the effect of the intra-band term of the conductivity (refer to Supplementary Note 1).

simulated transmission (in red) overlapping the experimental transmission curves of Fig. 1e (shown in black and blue colors for the forward and backward voltage sweeps, respectively). We emphasize that the simulated device has the same geometry as the real device (refer to Fig. 1e), meaning a width of the Si waveguide core of 750 nm, a core height of 220 nm, a cladding thickness of 10 nm, a device length of 60 μm, a bottom hBN thickness of 5 nm, a top hBN of 21 nm, a total hBN dielectric of 17 nm, a HfO_2 layer of 10 nm, a metal contact distance of 2680 nm, a metal contact thickness of 48 nm and a 450 nm-wide overlap region between the bottom and top graphene electrodes. We use a hBN relative permittivity of 3.8 (see Supplementary Note 5) but the HfO_2 permittivity has been set to 9.5 in order to reproduce the experimental transmission curves. This HfO_2 permittivity value (ϵ_{HfO_2}=9.5) has been used in all simulations in order to reproduce the measured transmission traces while keeping the dimensions and geometry of the simulated devices equal to the measured ones. The HfO_2 quality is expected to degrade when grown over hBN, it is therefore expected that the relative permittivity of HfO_2 grown over hBN is lower than stand-alone HfO_2 (Supplementary Note 5). We consider this simulation extracted ϵ_{HfO_2} as a valid evaluation of the HfO_2 relative permittivity when grown over hBN. For all the calculations throughout this work, we then consider the relative permittivity of HfO_2 over hBN to be ϵ_{HfO_2}=9.5.

We point out that the maximum voltage applied in Fig. 1e of the main text (or in Fig. S15) is $V_{BT} = 12.1\,\text{V}$, or equivalently E_F=0.41 eV (refer to Supplementary Note 7). This value is far from the breakdown voltage of the hBN-HfO_2-hBN dielectric (see Fig. 3c and Supplementary Note 5). To evaluate the E_F^max (i.e. the Fermi energy at the dielectric breakdown) for the device in Fig. 1e, we studied the dielectric characteristics of MIM capacitors with a hBN-HfO_2 dielectric combination (see Supplementary Note 5). The maximum breakdown field of the hBN-HfO_2 dielectric with a 10 nm thick HfO_2 and a 17 nm total hBN thickness (the same thicknesses as the modulator device in Fig. 1e) is $E_\text{BD} \approx 8.9\,\text{MV cm}^{-1}$ (see black dashed lines in Fig. S7 of Supplementary Note 5). This breakdown field corresponds to a maximum Fermi Energy of $E_\text{F}^\text{max} \approx 0.57$eV (also indicated by a black star in Fig. 3a of the main text), determined through the relation:

$$E_\text{F}^\text{max} = \hbar \nu_\text{F} \sqrt{\pi \epsilon_0 \epsilon_\text{rel} E_\text{BD}/q}, \tag{15}$$

where ϵ_rel is the equivalent dielectric constant of a hBN-HfO_2-hBN dielectric with a 10 nm-thick HfO_2 layer and total hBN thickness of 17 nm and considering the relative permittivity for hBN and HfO_2 as ϵ_hBN =3.8 and $\epsilon_{HfO_2} \approx$9.5, respectively (see Supplementary Note 7 for details). This value of $E_\text{F}^\text{max} \approx 0.57$eV corresponds to a $V_\text{BT}^\text{max} \approx 24\,\text{V}$ (see black vertical dashed line in Fig. S15) and determines a potential IL $\approx$ 0.042 dB considering the normalized transmission (right-axis in Fig. S15), i.e. neglecting the absorption due to polymer residues deposited along the waveguide during the fabrication processes and the fiber-to-fiber losses of a pristine waveguide.

If we include all the unwanted losses, i.e. we consider the measured raw transmission (left axis in Fig. S15), we obtain a measured IL $\approx$ 28.342 dB (Fig. S15). This value is in agreement with the transmission losses (27.75 dB) of a device-free Si-based optical waveguide that followed the same fabrication steps as the measured device. The IL of the modulator device in Fig. 1e is IL $\approx$ 7.8 dB (marked in blue in Fig. S15, measured ER $\approx$ 4.4 dB), with a potential IL as low as IL $\approx$ 0.042 dB (marked also in blue in Fig. S15), meaning a potential ER $\approx$ 12 dB. We note that this potential IL is achievable experimentally from the quality of our graphene devices and the robustness of the hBN-HfO_2-hBN dielectric.

Supplementary Note 11 $f_{-3\text{dB}}$ bandwidth

The electrical bandwidth $f_{-3\text{dB}}$ of the device can be determined as:

$$f_{-3\text{dB}} = \frac{1}{2\pi RC}, \tag{16}$$

where R and C are the total resistance and capacitance of the device.

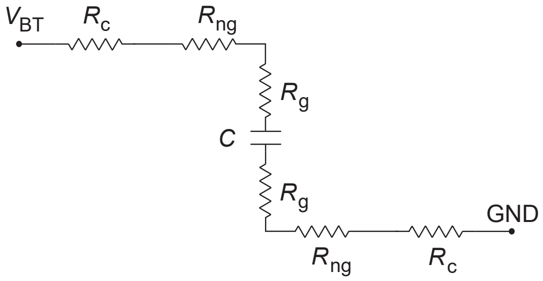

Supplementary Figure S16. Electrical circuit of the double-layer graphene modulator. Equivalent RC electronic circuit of the DL graphene EA modulator. V_{BT} is the input voltage, R_{c} the contact resistance, R_{ng} the non-gated graphene resistance and R_{g} the gated graphene resistance. C is the capacitance of the device and GND the ground.

In case of a DL graphene EA modulator (see Fig. S16), R can be defined as:

$$R = 2\,(R_{\text{c}} + R_{\text{ng}} + R_{\text{g}}), \tag{17}$$

where:

$$R_{\text{c}} = \frac{\rho_{\text{c}}}{L}, \tag{18}$$

$$R_{\text{ng}} = \frac{W_{\text{ng}}}{\sigma_{\text{ng}} L} \tag{19}$$

$$R_{\text{g}} = \frac{W_{\text{g}}}{2\sigma_{\text{g}} L} \tag{20}$$

where $\rho_{\text{c}} \approx 808.5$ Ω·µm is the average contact resistivity of the holes and electrons side (extracted from TLM measurements, see Supplementary Note 4) and $L \approx 60$ µm is the contact length of the device presented in Fig. 1. The width of the gated and non-gated regions are $W_{\text{g}} \approx 450$ nm and $W_{\text{ng}} \approx 2830$ nm, respectively. The non-gated conductivity ($\sigma_{\text{ng}} = n_{\text{ng}} q\mu$) considers the charge carrier density of the non-gate region (n_{ng}) evaluated at $E_{\text{F}} \approx 0.172$eV (intrinsic doping extracted from the hBN modulator in Supplementary Note 2). The gated conductivity ($\sigma_{\text{g}} = n_{\text{g}} q\mu$) considers the charge carrier density of the gated graphene region (n_{g}) evaluated at $V_{\text{BT}} = 10.4$ V (or equivalently $E_{\text{F}} = 376$ meV).

The capacitance C of the device includes three capacitance in series: the top layer graphene quantum capacitance ($C_{\text{Q}}^{\text{top}}$), the parallel plate capacitance (C_{ox}), and the bottom layer graphene quantum capacitance ($C_{\text{Q}}^{\text{bottom}}$) [6] [20]:

$$\frac{1}{C} = \frac{1}{C_{\text{Q}}^{\text{top}}} + \frac{1}{C_{\text{ox}}} + \frac{1}{C_{\text{Q}}^{\text{bottom}}} \tag{21}$$

where C_{Q} is defined as

$$C_{\text{Q}} = \frac{2W_{\text{g}} L q k_{\text{B}} T}{\pi(\hbar\nu_{\text{F}})^2} \log\left[2\left(1 + \cosh\frac{E_{\text{F}}}{k_{\text{B}}T}\right)\right] \tag{22}$$

where E_F = 376 meV (Pauli blocking), W_g = 450 nm corresponds to the overlap region between the top and bottom graphene electrodes, and

$$C_\mathrm{ox} = \frac{W_\mathrm{g} L \epsilon_0 \epsilon_\mathrm{r}}{d} \tag{23}$$

with ϵ_0 is vacuum permittivity, $d = d_\mathrm{hBN} + d_{\mathrm{HfO}_2}$ and ϵ_r is defined as:

$$\epsilon_\mathrm{r} = (d_\mathrm{hBN} + d_{\mathrm{HfO}_2}).(\frac{d_\mathrm{hBN}}{\epsilon_\mathrm{hBN}} + \frac{d_{\mathrm{HfO}_2}}{\epsilon_{\mathrm{HfO}_2}})^{-1} \tag{24}$$

where d_hBN = 17 nm, d_{HfO_2} = 10 nm, ϵ_hBN = 3.8, $\epsilon_{\mathrm{HfO}_2}$ = 9.5 (see Supplementary Note 5 and Supplementary Note 10).

Supplementary Note 12 High-frequency measurements setup

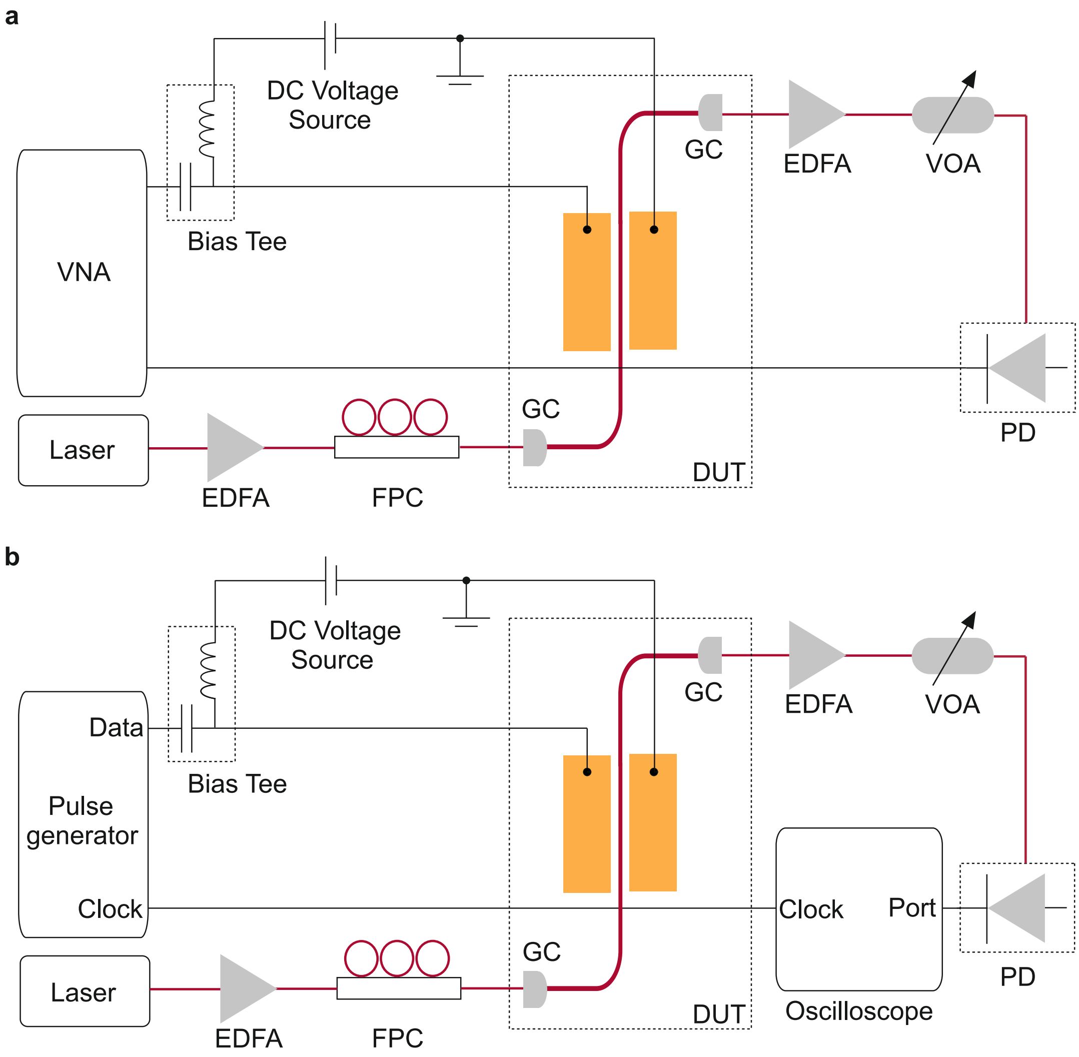

Supplementary Figure S17. High frequency measurement setups to measure frequency response (panel a), and eye diagram (panel b). Set-up for measuring the EA modulator electro-optic response of figure 2b of the main text up to 40 GHz (Vector network analyzer (VNA) limited). The optical path (in red) is composed of a laser source (λ = 1550 nm), an erbium-doped fiber amplifier (EDFA), a fiber-polarization controller (FPC), a variable optical attenuator (VOA) and a high-speed photodetector (PD). The device under test (DUT) consists of two grating couplers (GC), the optical waveguide and the graphene EAM (refer to figure 1 of the main text). The electronic path (in black) connects VNA, DC voltage source and device through a bias tee.

Supplementary Note 13 Frequency response measurements

We recorded a second frequency response measurement (scan 2 in Fig. S18) from the device in Fig. 1. Both 1 and 2 scans (scan 1 corresponds to the frequency response in Fig. 2b of the main text) have been measured with no temporal averaging and a low intermediate frequency filtering (IF BW) of 1kHz to 10KHz. Both measurements (Fig. S18) are without de-embedding (i.e. without subtracting the contribution from setup).

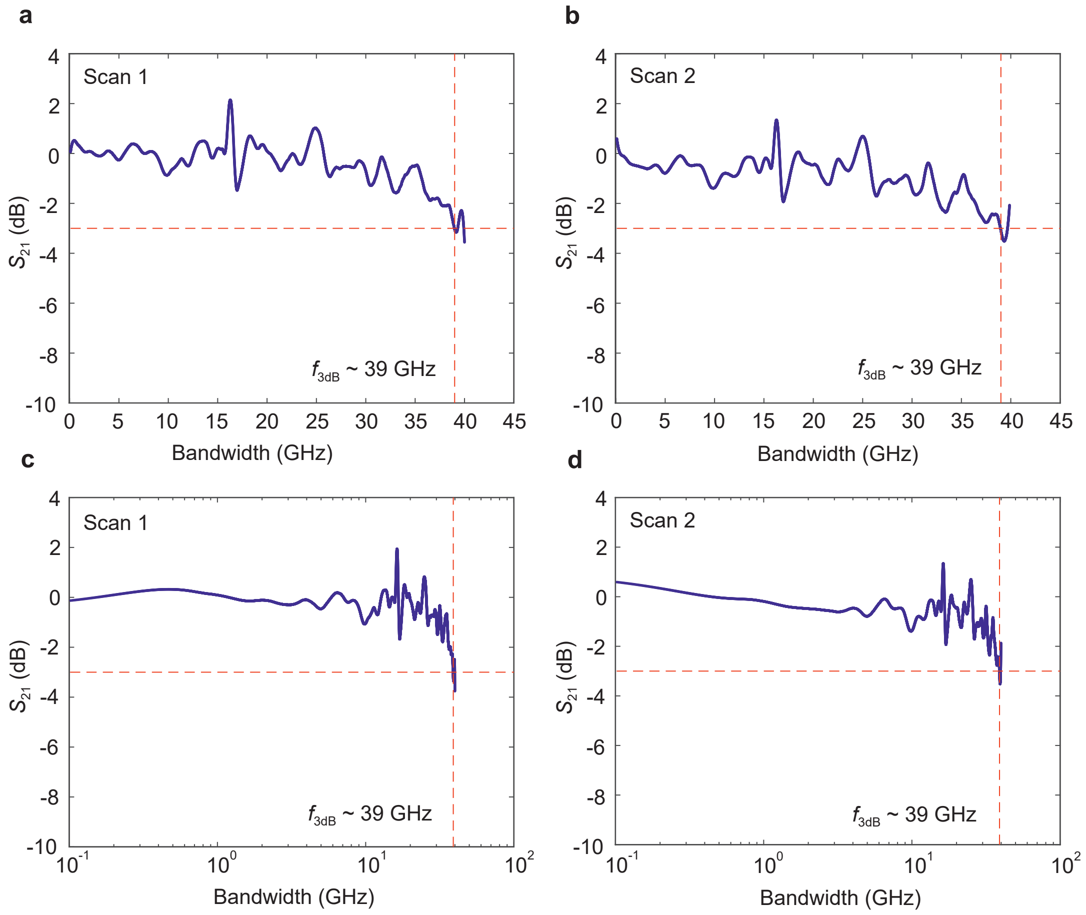

Supplementary Figure S18. Bandwidth analysis. Two different frequency response measurements (**a-c** and **b-d**) of the same device as in Fig. 1 and 2 of the main text, represented in a linear and a log scale (panels **a-b** and **c-d**, respectively).

Supplementary Note 14 10 Gbps eye diagram measurement

We also measured our device at lower speeds. Fig. S19 is an eye-diagram measured at 10 Gbps for the device presented in Fig. 1, and 2 of the main text.

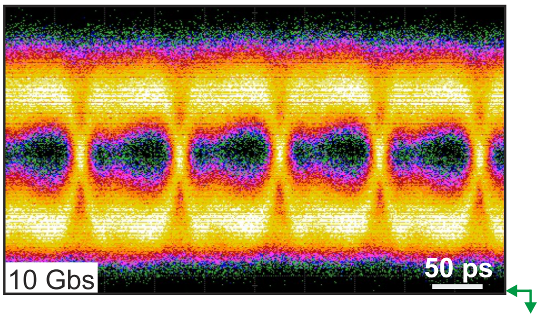

Supplementary Figure S19. Eye-diagram of the device in Fig. 1 of the main text. The eye-diagram has been measured at 10Gbps (SNR=2.1dB). The green arrow indicates the *0W* baseline.

Supplementary Note 15 Comparison of graphene-based EA modulators

Supplementary Table 2. Static characterization comparison for graphene-based EA modulators.

Reference	Wavelength (nm)	Modulation efficiency (dB V^{-1})	Extinction ratio ER (dB)	Insertion Loss IL (dB)	ER/IL
Single-layer graphene					
[23]	1530	1.2*	4	NA	NA
[14]	1550	1.22*	5.2	3.8 †	1.36 †
[24]	1550	1	3.5	NA	NA
[25]	1550	0.44*	1.2	40 ‡	0.03 ‡
Double-layer graphene					
[26]	1537	1.5*	6.5	4 †	1.625 †
[27]	1550	0.13*	2	0.9 ‡	2.22 ‡
[28]	1550	1.42*	16	3.3 †	4.8 †
[29]	1550	0.4*	1.4	NA	NA
[22]	1550	1.37*	3	13 ‡	0.23 ‡
Dev. in Fig. 1e (L.B.)	1550	2.2*	4.4	7.8 ‡	0.57 ‡
Dev. in Fig. 1e (U.B.)	1550	2.2*	12	0.042 ‡	285.71 ‡
Dev. in Fig. 3c	1550	1.3*	7.8	0.04 ‡	195 ‡

Note:

* These values are extracted from a linear fit in a 0.5V interval.

† The reported IL and ER/IL values include the fabrication impurities deposited along the waveguide as well as non-uniform charge distribution in the graphene and hence, cannot be directly compared to our values.

‡ The reported IL and ER/IL values are attributed to the graphene EA modulator device itself.

- The calculation of the ER/IL lower bound (LB) for device in Fig. 1e considers the measured ER $\approx$ 4.4 dB and IL $\approx$ 7.8 dB. The upper bound (UB) considers the potential ER $\approx$ 12 dB and IL $\approx$ 0.042 dB (refer to Supplementary Note 10for details).
- Both devices in Figs. 1e and 3c are based on a hBN-HfO_2-hBN dielectric.

Supplementary Table 3. Dynamic characterization comparison for graphene-based EA modulators.

Reference	Footprint (μm^2)	EO bandwidth f_{3dB} (GHz)	Maximum bit rate (Gbps)	Power consumption (fJ bit^{-1})	Dynamic ER (dB)	Normalized dynamic ER (dB μm^{-1})
Single-layer graphene						
[23]	25	1.2	NA	NA	NA	NA
[14]	37.5	5.9	10	350	2.3 @2.5 V	0.046
[24]	48	5	10	NA	1.4 @1.1 V	0.014
[25]	20	NA	NA	NA	NA	NA
Double-layer graphene						
[26]	40	1	NA	1000	NA	NA
[27]	18	35	NA	1400	NA	NA
[28]	150	0.67	NA	NA	NA	NA
[29]	25	NA	NA	NA	NA	NA
[22]	78	30	50	NA	1.3 @3.5 V	0.011
Dev. in Fig. 1e	27	> 39	40	159.9	5.2 @3.5 V	0.086

Supplementary Note 16 Optical power penalty calculations

We calculated the optical power penalty (on-state loss, loss due to on/off and eye-closure penalty) of the modulators in Fig. 1e and Fig. 3c of the main text. The calculations are based on ref. [30, 31] and are summarized in Table 4 together with the comparison with state-of-the-art single- and double-layer graphene modulators.

The upper bound of the optical power penalty (OPP) for the device in Fig. 1e (marked UB in Table 4) has been calculated from the measured ER and IL (see Supplementary Note 10). The lower bound has been evaluated considering the potential IL derived in Supplementary Note 10 (IL≈ 0.042 dB). We emphasize that the reported lower bound for the OPP value is the lowest reported value so far, amongst all graphene-based modulators (refer to Table 4).

Supplementary Table 4. Penalty calculations for graphene-based EA modulators.

Reference	On-state loss	Loss due to on/off	Eye-closure penalty	OPP* (dB)
Single-layer graphene				
[14]	0.126 †	2.16 †	-0.536 †	8.37 †
[25]	∼0 ‡	1.16 ‡	-0.137 ‡	49.2 ‡
Double-layer graphene				
[26]	0.089†	2.73 †	-0.634 †	8.11 †
[27]	0.513 ‡	1.29 ‡	-0.226 ‡	8.24 ‡
[28]	0.012 †	20.4 †	-0.951 †	6.42 †
[22]	0.025 ‡	1.5 ‡	-0.332 ‡	19.0 ‡
Dev. in Fig. 1e (UB)	0.06 ‡	1.89 ‡	-0.47 ‡	12.7 ‡
Dev. in Fig. 1e (LB)	0.0625 ‡	8.42 ‡	-0.88 ‡	3.34 ‡
Dev. in Fig. 3c	0.164 ‡	3.51 ‡	-0.72 ‡	3.84 ‡

Note:

* The optical power penalty is defined as OPP = $(P_{\text{out}}(1)$ - $P_{\text{out}}(0))/(2$ x $P_{\text{in}})$, where $P_{\text{out}}(1)$, and $P_{\text{out}}(0)$ are the high and low levels of the optical power output extracted from the ER and IL during static measurements. P_{in} is the input optical power [30, 31].

† The reported IL and ER/IL values include the impurities from the fabrication along the waveguide as well as non-uniform charge distribution in the graphene and hence, cannot be directly compared to our values.

‡ The reported IL and ER/IL values are attributed to the graphene EA modulator device itself.

- The calculation of the upper bound (UB) for the OPP of device in Fig. 1e considers the total measured IL≈ 7.8 dB, whereas the lower bound (LB) is calculated using the potential IL≈ 0.042 dB (Supplementary Note 10).

References

[1] GoncÌğalves, P. A. D. & Peres, N. M. R. An introduction to graphene plasmonics. *World scientific, Singapore* (2016).

[2] Hanson, G. W. Dyadic Green's functions and guided surface waves for a surface conductivity model of graphene. *J. Appl. Phys.* **103** (2008).

[3] Sarma, S. D., Adam, S. & Rossi, E. Electronic transport in two-dimensional graphene. *Rev. Mod. Phys.* **83** (2011).

[4] Yan, J., Zhang, Y., Kim, P. & Pinczuk, A. Electric field effect tuning of electron-phonon coupling in graphene. *Phys. Rev. Lett.* **98**, 166802 (2007).

[5] Novoselov, K. S. *et al.* Electric field effect in atomically thin carbon films. *Science* **306**, 666–669 (2004).

[6] Xia, J., Chen, F., Li, J. & Tao, N. Measurement of the quantum capacitance of graphene. *Nature Nanotechnology* **4**, 505–509 (2009).

[7] Pizzocchero, F. *et al.* The hot pick-up technique for batch assembly of van der Waals heterostructures. *Nature Communications* **7**, 11894 (2016).

[8] Purdie, D. G. *et al.* Cleaning interfaces in layered materials heterostructures. *Nature Communications* **9**, 5387 (2018).

[9] Lee, J. E., Ahn, G., Shim, J., Lee, Y. S. & Ryu, S. Optical separation of mechanical strain from charge doping in graphene. *Nature Communications* **3**, 1024 (2012).

[10] Kim, S. *et al.* Realization of a high mobility dual-gated graphene field-effect transistor with Al_2O_3 dielectric. *Applied Physics Letters* **94**, 062107 (2009).

[11] Hwang, E. H., Adam, S. & Sarma, S. D. Carrier transport in two-dimensional graphene layers. *Phys. Rev. Lett.* **98** (2007).

[12] Yan, J., Zhang, Y., Kim, P. & Pinczuk, A. Electric field effect tuning of electron-phonon coupling in graphene. *Phys. Rev. Lett.* **98** (2007).

[13] Falkovsky, L. A. Optical properties of graphene and IV–VI semiconductors. *Phys.-Usp.* **51**, 887–897 (2008).

[14] Hu, Y. *et al.* Broadband 10 Gbps operation of graphene electro-absorption modulator on silicon. *Laser & Photonics Reviews* **10**, 307–316 (2016).

[15] Wang, L. *et al.* One-dimensional electrical contact to a two-dimensional material. *Science* **342**, 614–617 (2013).

[16] Yota, J., Shen, H. & Ramanathan, R. Characterization of atomic layer deposition HfO_2, Al_2O_3, and plasma-enhanced chemical vapor deposition Si_3N_4 as metal-insulator-metal capacitor dielectric for GaAs HBT technology. *Journal of Vacuum Science & Technology A* **31**, 1–9 (2013).

[17] Hattori, Y., Taniguchi, T., Watanabe, K. & Nagashio, K. Anisotropic dielectric breakdown strength of single crystal hexagonal boron nitride. *ACS Appl. Mater. Interfaces* **8**, 27877–27884 (2016).

[18] Hattori, Y., Taniguchi, T., Watanabe, K. & Nagashio, K. Layer-by-layer dielectric breakdown of hexagonal boron nitride. *ACS Nano* **9**, 916–921 (2015).

[19] Lukosius, M. *et al.* Atomic-vapour-deposited HfO_2 and $Sr_4Ta_2O_9$ layers for metal-insulator-metal applications. *Microelectronic Engineering* **84**, 2165–2168 (2007).

[20] Sorianello, V., Midrio, M. & Romagnoli, M. Design optimization of single and double layer graphene phase modulators in SOI. *Opt. Express* **23**, 6478–6490 (2015).

[21] Romagnoli, M. *et al.* Graphene-based integrated photonics for next-generation datacom and telecom. *Nature Reviews Materials* **3**, 392–414 (2018).

[22] Giambra, M. A. *et al.* High-speed double layer graphene electro-absorption modulator on SOI waveguide. *Opt. Express* **27**, 20145–20155 (2019).

[23] Liu, M. *et al.* A graphene-based broadband optical modulator. *Nature* **474**, 64–67 (2011).

[24] Sorianello, V. *et al.* Chirp management in silicon-graphene electro absorption modulators. *Opt. Express* **25**, 19371–19381 (2017).

[25] Kou, R. *et al.* Ultra-fine metal gate operated graphene optical intensity modulator. *Appl. Phys. Lett.* **109**, 1–6 (2016).

[26] Liu, M., Yin, X. & Zhang, X. Double-layer graphene optical modulator. *Nano Lett.* **12**, 10–13 (2012).

[27] Dalir, H., Xia, Y., Wang, Y. & Zhang, X. Athermal broadband graphene optical modulator with 35 GHz speed. *ACS Photonics* **3**, 1564–1568 (2016).

[28] Mohsin, M. *et al.* Graphene based low insertion loss electro-absorption modulator on SOI waveguide. *Optics Express* **22**, 15292–15297 (2014).

[29] Kleinert, M. *et al.* Graphene-based electro-absorption modulator integrated in a passive polymer waveguide platform. *Optical Materials Express* **6**, 1800–1807 (2016).

[30] Li, G. *et al.* Ring resonator modulators in silicon for interchip photonic links. *IEEE Journal of Selected Topics in Quantum Electronics* **19**, 95–113 (2013).

[31] Srinivasan, S. A. *et al.* 56 Gbps germanium waveguide electro-absorption modulator. *Journal of Lightwave Technology* **34**, 419–424 (2016).
